# Factors that influence women's engagement with breastfeeding support: A qualitative evidence synthesis

**DOI:** 10.1111/mcn.13405

**Published:** 2022-08-25

**Authors:** Theresa Bengough, Shoba Dawson, Hui‐Lin Cheng, Alison McFadden, Anna Gavine, Rebecca Rees, Emma Sacks, Karin Hannes

**Affiliations:** ^1^ Research Group SoMeTHin'K (Social, Methodological and Theoretical Innovation/Kreative), Faculty of Social Sciences KU Leuven Leuven Belgium; ^2^ National Centre for Early Childhood Intervention The Austrian Public Health Institute Vienna Austria; ^3^ Faculty of Health Sciences, Bristol Medical School University of Bristol Bristol UK; ^4^ School of Nursing The Hong Kong Polytechnic University Kowloon Hong Kong; ^5^ School of Health Sciences University of Dundee Dundee Scotland UK; ^6^ EPPI‐Centre, Social Science Research Unit, UCL Institute of Education University College London London UK; ^7^ Department of International Health Johns Hopkins Bloomberg School of Public Health Baltimore Maryland USA

**Keywords:** breastfeeding, breastfeeding support, contextual factors, early childhood interventions, policy and practice, qualitative systematic synthesis

## Abstract

Breastfeeding is an integral part of early childhood interventions as it can prevent serious childhood and maternal illnesses. For breastfeeding support programmes to be effective, a better understanding of contextual factors that influence women's engagement and satisfaction with these programmes is needed. The aim of this synthesis is to suggest strategies to increase the level of satisfaction with support programmes and to better match the expectations and needs of women. We systematically searched for studies that used qualitative methods for data collection and analysis and that focused on women's experiences and perceptions regarding breastfeeding support programmes. We applied a maximum variation purposive sampling strategy and used thematic analysis. We assessed the methodological quality of the studies using a modified version of the CASP tool and assessed our confidence in the findings using the GRADE‐CERQual approach. We included 51 studies of which we sampled 22 for in‐depth analysis. Our sampled studies described the experiences of women with formal breastfeeding support by health care professionals in a hospital setting and informal support as for instance from community support groups. Our findings illustrate that the current models of breastfeeding support are dependent on a variety of contextual factors encouraging and supporting women to initiate and continue breastfeeding. They further highlight the relevance of providing different forms of support based on socio‐cultural norms and personal backgrounds of women, especially if the support is one‐on‐one. Feeding decisions of women are situated within a woman's personal situation and may require diverse forms of support.

## Key messages

- Supporting women to initiate and continue to breastfeed is a complex process.
- The current models of breastfeeding support are dependent on a variety of contextual factors encouraging and supporting women to initiate and continue breastfeeding.
- Practitioners and service developers need to be aware that throughout the whole continuum of maternity care, women's breastfeeding support needs are dynamic, and it is unpredictable when challenges and needs arise.
- The emotional and physical difficulties sometimes associated with breastfeeding may require diverse forms of support, also in combination, to counteract challenges.

## 1 INTRODUCTION

There are numerous health benefits of breastmilk on child health, such as reduced risk of hospitalisation due to respiratory tract infections, otitis media and of Sudden Infant Death Syndrome, as well as reduced incidence of gastrointestinal tract infections, clinical asthma and atopic dermatitis (Brown et al., 2020; McFadden et al., 2016; Rollins et al., 2016). Additionally, obesity rates are significantly lower in children, adolescents, and adults who have been breastfed (Victora et al., 2016; WHO, 2016). The short‐ and long‐term benefits for breastfeeding women include decreased postpartum blood loss and more rapid involution of the uterus, rapid weight loss, a decreased risk of type 2 diabetes mellitus and breast cancer (Victora et al., 2016; Buckland et al. 2020). Crucially, there is a dose–response relationship for many of these outcomes, with the greatest benefits being realised when exclusive breastfeeding is practised for 6 months (Kramer & Kakuma, 2012). The cost‐effectiveness of breastfeeding has been highlighted (Pokhrel, 2015). The prevalence of not breastfeeding is associated with global financial losses of about $302 billion annually (Rollins et al., 2016). Consequently, different stakeholders have substantial interest in the promotion, protection, and support of breastfeeding. The World Health Organization (WHO) presents optimal breastfeeding as one of the most effective interventions in achieving maternal and child health and advises a period of exclusive breastfeeding of up to 6 months (World Health Organization, 2017).

Despite the numerous benefits identified, initiation rates could be increased. Even when breastfeeding is initiated, continuation rates at an international level are low (World Health Organization, 2017). In most countries, rates of exclusive breastfeeding at 6 months are below 50%. In low‐ and middle‐income countries (LMICs) whilst most women practise some breastfeeding at 6 months, only 35% exclusively breastfeed under 6 months. In high‐income countries (HICs) few women exclusively breastfeed at 6 months, despite over 75% of women initiating breastfeeding (Victoria et al., 2016). However, even within HIC and LMICs rates of breastfeeding vary, for instance, the prevalence of breastfeeding at 12 months is <1% in the United Kingdom and is 35% in Norway (Victora et al., 2016). In addition, rates of breastfeeding also vary within countries according to socio‐demographic characteristics. Within HICs breastfeeding rates were higher among high‐income, better educated women compared to women from lower‐income groups (Victoria et al., 2016). Conversely, in LMICs breastfeeding rates were higher in women from lower‐income groups compared to women from high‐income groups. Also, women from other vulnerable groups (e.g., teenage mothers), have lower breastfeeding rates (Buckland et al., 2020). Despite this, with effective support, women from vulnerable groups can breastfeed exclusively and for longer periods (McFadden et al., 2019).

When women are asked about why they stop breastfeeding, the following reasons are often cited: fatigue, inconvenience, concerns about milk supply, painful nipples or poor guidance from health care professionals (Amir et al., 2021). All these may result in early discontinuation of breastfeeding (Demirci & Bogen, 2017; Li et al., 2008) and can often be rectified with skilled support to enable women to continue breastfeeding (McFadden et al., 2019). Such interventions tend to have a positive impact on both the initiation and continuation of breastfeeding (Fewtrell et al., 2020; McFadden et al., 2017). For example, they may influence women's resilience towards their environment by providing them with meaningful resources to position themselves in society or challenge the thoughts and prejudices of their partners and family.

Despite findings from meta‐analyses suggesting that overall breastfeeding support interventions can improve breastfeeding rates (Beake et al., 2012; McFadden et al., 2017), there is considerable statistical heterogeneity in the study results and clinical heterogeneity in terms of the interventions and participants. There is therefore a need to better understand why support interventions succeed or fail. Qualitative studies generally give a voice to those who experience an intervention or life situation, allowing them to share concerns and suggestions that relate to their specific needs. Such insights might help develop support programmes that better match the needs of women. Evidence from qualitative studies can provide valuable insights into barriers and facilitators experienced by women associated with suboptimal uptake, initiation and continuation, and engagement and satisfaction with support programmes. It may further improve the equity of programmes by understanding who is not benefitting and why. In this qualitative synthesis barriers and facilitators are understood as contextual factors, including personal, cultural, political, social, psychological and other factors that impact on women's engagement or responsiveness to support as well as factors that influence their overall satisfaction with support (Bengough et al., 2018).

### 1.1 Why is it important to do this evidence synthesis?

While several qualitative syntheses and quantitative reviews exploring barriers and facilitators towards breastfeeding have been published, they have a different focus, target group, or cover different time spans compared to ours. Differences in focus are outlined in Table 1 where we focused on reviews with a similar conceptualisation of support interventions.

**Table 1:** Summary of relevant published (Cochrane) reviews (qualitative and quantitative)

Author(s)	Title	A comparison of this review with other reviews	Methodology
**Qualitative and quantitative reviews directly relevant for the QES**			
Leeming et al. (2022)	Self‐conscious emotions and breastfeeding support: A focused synthesis of UK qualitative research	**What do they do**: Leeming et al. examined qualitative research reporting women's perspectives on receiving formalised breastfeeding support to understand whether and how self‐conscious emotions are relevant to interactions with those providing support. **Results**: The review concluded that self‐conscious emotions and related self‐appraisals, particularly negative ones, are relevant to many UK women's experiences of varied types of breastfeeding support. Many women can find their mothering identity precarious and in need of active management when interacting with supporters. Careful emotion work is required to avoid feeling guilt, shame, embarrassment or even humiliation. **What do we add**: This review limited the context to evidence from the United Kingdom, focuses on support implemented in the postnatal phase and included women with a healthy term baby only. It has therefore a narrower focus than our QES. Additionally, it excluded online support via social media. In our QES we acknowledge studies on online support which, due to recent Covid‐19 climates, will warrant increasing attention in the area of breastfeeding support.	Qualitative
Priscilla et al. (2021)	A Qualitative Systematic Review of Family Support for a Successful Breastfeeding Experience among Adolescent Mothers	**What do they do**: Priscilla et al. synthesise, and recognise the qualitative evidence of family support for successful breastfeeding among teen mothers. **Results**: The review founds that family significantly affect the success of breastfeeding practices among adolescent mothers through their appraisal, instrumental, emotional, and informational support. The family strengthens the adolescence's breastfeeding decisions and confidence, provide financial assistance, share positive breastfeeding information and experience, encourage them to continue the feeding and motivate theme to pursue their study. **What do we add**: This review includes studies on informal family support for breastfeeding women and therefore has a narrower focus, as it excludes studies of formal support. Additionally, it focuses only on adolescent mothers and excluded studies that focused on other types of mothers.	Qualitative
Allen et al. (2021)	Avoidance of bottles during the establishment of breastfeeds in preterm infants.	**What do they do**: Allen et al. identified the effects of avoidance of bottle feeds during establishment of breastfeeding on the likelihood of successful breastfeeding, and assessed the safety of alternatives to bottle feeds. **Results**: Avoiding the use of bottles when preterm infants need supplementary feeds probably increases the extent of any breastfeeding at discharge and may improve any and full breastfeeding (exclusive) up to 6 months postdischarge. Most of the evidence demonstrating benefit was for cup feeding. The authors express uncertainty whether a tube alone approach to supplementing breastfeeds improves breastfeeding outcomes; further studies of high certainty are needed to determine this. As breastfeeding support happens in the context of human action and interaction in this review, it becomes directly relevant for our QES. **What do we add**: Our QES could give important insights in the type of information relevant for mothers but also on the channel of information. It would allow to identify process and implementation factors that may need to be considered in the role out of support, particularly in relation to the evidence on why it is beneficial for mother and infant to breastfeed. The findings of the QES can assist in identifying outcome measures that mothers perceive as important to consider in future updates of the review.	Quantitative
Buckland et al. (2020)	Interventions to promote exclusive breastfeeding among young mothers: a systematic review and meta‐analysis	**What do they do**: Buckland et al. examined the range and effectiveness of interventions which have been designed to increase rates of EBF among young mothers in high‐income countries. **Results**: The interventions included peer counselling, telephone support, massage, gift packs, financial incentive and antenatal education. Most studies included a combination of strategies, peer counselling being the most common. A meta‐analysis of four of nine included studies did not detect a difference in rate of exclusive breastfeeding to 3 months postpartum. **What do we add**: Our QES may be able to generate data on the relevance or non‐relevance of providing different types of support in different geographical locations or settings as this review focuses on high‐income countries only, whereas our QES does not focus on one particular setting. It could further provide an in‐depth insight into factors that influence the needs of the particular target group of young mothers. The findings of the QES can assist in identifying outcome measures that young mothers perceive as important to consider in future updates of the review.	Quantitative
Whitford et al. (2017)	Breastfeeding education and support for women with twins or higher‐order multiples.	**What do they do**: Whitford et al. assessed the effectiveness of breastfeeding education and support for women with twins or higher‐order multiples. **Results**: Support and education about breastfeeding have been found to improve the duration of any breastfeeding for healthy term infants and their mothers, however evidence is lacking about interventions that are effective to support women with twins or higher‐order multiples. None of the interventions described in the identified studies were specifically designed for women with more than one infant. As breastfeeding support happens in the context of human action and interaction in this review, it becomes directly relevant for our QES. **What do we add**: Our QES could give important insights in the type of human support these mothers need, compared to those with only one infant. It would allow to identify process and implementation factors that may need to be considered in the role out of educational support for this particular target group, particularly in relation to the evidence on optimal delivery modes, timing of the intervention, or staff requirements. The findings of the QES can assist in identifying outcome measures that mothers with twins or multiples perceive as important to consider in future updates of the review.	Quantitative
McFadden et al. (2017)	Support for healthy breastfeeding mothers with healthy term babies.	**What do they do**: McFadden et al. assessed the effectiveness of different modes of offering similar supportive interventions and whether interventions containing both antenatal and postnatal elements were more effective than those taking place in the postnatal period alone. Additionally, they examined the effectiveness of different care providers and training and the impact of setting and/or timing of interventions as well as the interaction between background breastfeeding rates and effectiveness of support.	Quantitative
		**Results**: When breastfeeding support is offered to women, the duration and exclusivity of breastfeeding are increased. Characteristics of effective support include: that it is offered as standard by trained personnel during antenatal or postnatal care, that it includes ongoing scheduled visits so that women can predict when support will be available, and that it is tailored to the setting and the needs of the population group. Support is likely to be more effective in settings with high initiation rates. Support may be offered either by professional or lay/peer supporters, or a combination of both. Strategies that rely mainly on face‐to‐face support are more likely to succeed with women practising exclusive breastfeeding.	
		**What do we add**: McFadden and colleagues investigated substantial heterogeneity for all four outcomes with subgroup analyses for the following covariates: who delivered care, type of support, timing of support, background breastfeeding rate and number of postnatal contacts. The findings of our QES may help to explain part of this heterogeneity by looking at contextual issues that potentially influenced the process or implementation of the intervention. The QES may also be able to generate data on the relevance or non‐relevance of providing different types of support in different geographical locations or to different populations and provide an in‐depth insight into factors that influence the needs of particular target groups.	
Balogun et al. (2016)	Interventions for promoting the initiation of breastfeeding.	**What do they do**: This Cochrane review examines the effectiveness of different types of breastfeeding promotion activities, in terms of changing the number of women who initiate breastfeeding as well as to evaluate the effectiveness of different types of breastfeeding promotion activities, in terms of changing the number of women who initiate breastfeeding early (within 1 h after birth). Furthermore, Balogun et al aimed at describing health promotion activities intended to increase the initiation rate of breastfeeding.	Quantitative
		**Results**: The authors concluded that support interventions (health care professional‐led breastfeeding education and non‐health care professional‐led counselling and peer support interventions) showed some improvements in terms of numbers of women initiating breastfeeding. However, due to the low quality of the studies, these findings should be interpreted with caution.	
		**What do we add**: The majority of the trials in Balogun's review were conducted in the USA, among women on low incomes and who varied in ethnicity and feeding intention, thus limiting the generalisability of these results to other settings. This is due to expected variety in ethnicity, income levels and feeding intentions of women from low‐ and middle‐income countries. In the absence of studies measuring the effectiveness of breastfeeding promotion activities in low‐ and high‐income settings our QES can help to identify contextual factors that may need to be considered to secure an efficient role out of the promotion programmes, taking into account process and implementation characteristics such as timeframes for delivery, how to reach out to these mothers and which specific components of the standard programmes need to be adapted to better fit the characteristics of the setting and target population for a large scale role out.	
Lumbiganon et al. (2016)	Antenatal breastfeeding education for increasing breastfeeding duration.	**What do they do**: Lumbiganon et al. evaluated the effectiveness of antenatal breastfeeding education for increasing breastfeeding initiation and duration. **Results**: There was no conclusive evidence in favour of any antenatal breastfeeding education for improving initiation of breastfeeding, proportion of women giving any breastfeeding or exclusively breastfeeding at 3 or 6 months or the duration of breastfeeding. The authors further state that the evidence in this review is primarily relevant to high‐income settings. **What do we add**: The QES would allow us to follow up on the limited evidence found for the effectiveness of the interventions researched. The QES could focus on experiences with these interventions to evaluate whether the non‐effectiveness is more likely related to the intrinsic qualities of the programmes, or contextual influences that may help explain their limited success rate.	Quantitative
Beake et al. (2012)	A systematic review of structured compared with nonstructured breastfeeding programmes to support the initiation and duration of exclusive and any breastfeeding in acute and primary health care settings.	**What do they do**: The objective of this review was to consider evidence of outcomes of structured compared with nonstructured breastfeeding programmes in acute maternity care settings to support initiation and duration of exclusive breastfeeding. **Results**: Beake et al. concluded that initiation and duration of exclusive breastfeeding and any other type of breastfeeding were positively influenced by structured programmes. These performed better than standard care and are recommended as being beneficial in health care settings with low breastfeeding initiation and duration rates.	Quantitative, Qualitative
		**What do we add**: Beake and colleagues considered quantitative as well as qualitative studies but did not include any qualitative studies after applying a quality appraisal exercise. We examined these qualitative studies for relevant findings regarding support programmes.	
Jolly et al. (2012)	Systematic review of peer support for breastfeeding continuation: meta‐regression analysis of the effect of setting, intensity, and timing.	**What do they do**: The aim of this review is to examine the effect of setting, intensity, and timing of peer support on breastfeeding. **Results**: The authors concluded that peer support interventions increased breastfeeding continuation in low‐ or middle‐income countries (mainly exclusive breastfeeding). However, this did not seem to apply in high‐income countries (particularly the UK). Peer support of low intensity did not prove to be effective. **What do we add**: Jolly et al. considered peer support programmes only. Our definition of support programmes is broader and includes various dimensions (see Table 2). We examined included studies for relevant qualitative evidence as well as considered interventions and outcome measures reported on.	Quantitative
Burns (2010)	A meta‐ethnographic synthesis of women's experience of breastfeeding.	**What do they do**: Burns and colleagues aimed at understanding the overall social phenomenon of breastfeeding rather than breastfeeding support. They compiled commonly used metaphors, ideas and phrases across the national and international qualitative studies. **Results**: The findings from this meta‐ethnographic synthesis have highlighted the contribution of sociocultural discourses to the sense of disillusionment, and failure that many women express. It is also evident that the words and language health professionals and other support people use may be contributing to the lack of confidence, and sense of guilt and failure that some new mothers report. **What do we add**: Burns and colleagues focus on the overall experience of women with breastfeeding rather than the experience with breastfeeding support programmes. We examined this review to inform our discussion section.	Qualitative
Schmied et al. (2009)	Women's perceptions and experiences of breastfeeding support: a meta‐synthesis.	**What do they do**: The primary aim of this meta‐synthesis was to examine women's perceptions and experiences of breastfeeding support, either professional or peer. The secondary aim was to highlight any differences between components of peer and professional support. **Results**: The findings of this review highlight the importance of person‐centred communication skills and of relationships in supporting women to breastfeed. Key elements of an authentic presence are trusting relationship, empathy, listening and responding to woman's needs. Within an organisation, programmes that focus on continuity of midwifery care or peer support models, are more likely to promote an authentic presence.	Qualitative
		**What do we add**: This review includes studies on formal or peer and professional support for breastfeeding women but has a narrower focus, as it excludes studies of family or informal support. Additionally, it focuses only on initiation of breastfeeding and excluded studies that focused on specific clinical subgroups, such as women post‐caesarean. To our knowledge, Schmied et al's review has not been updated with studies published after the year 2007. We examined the review's qualitative studies to select those that match our inclusion criteria.	
McInnes and Chambers (2008)	Supporting breastfeeding mothers: qualitative synthesis.	**What do they do**: The aim of this synthesis was to examine mothers' and health care professionals' experiences and perceptions of breastfeeding support. **Results**: Mothers tended to rate social support of more important than health service support. This was due to factors like time pressure, lack of availability of health care professionals or guidance (amongst others). The authors further suggest addressing the needs, both of mothers and staff when changing health services. **What do we add**: This review is limited in the type of participants included. It does not address the concerns of mothers‐to‐be. Instead, it includes perspectives of health care professionals. To our knowledge, McInnes and Chamber's review has not been updated with studies published after the year 2007. We examined the review's qualitative studies to select those that match our inclusion criteria.	Qualitative
Fairbank et al. (2000)	A systematic review to evaluate the effectiveness of interventions to promote the initiation of breastfeeding.	**What do they do**: Fairbank and colleagues aimed at evaluating existing evidence to identify which promotion programmes are effective at increasing the number of women who start to breastfeed. In addition, the review aimed to assess the impact of such programmes on the duration and/or exclusivity of breastfeeding and the intermediate and process outcomes. The authors also considered to explore effects on women from different socioeconomic backgrounds.	Quantitative
		**Results**: Interventions, delivered as a stand‐alone intervention in developed countries, proved to be effective. Informal, small group health education delivered during the antenatal period, seemed to be effective at increasing initiation rates. One‐to‐one health education appears to be effective at increasing initiation rates among women on low incomes. Peer support programmes, delivered in the ante‐ and postnatal periods, showed to be effective at increasing initiation and duration rates of breastfeeding among women on low incomes, and particularly among women who are in favour of breastfeeding.	
		**What do we add**: This review has a quantitative focus. Since we defined women from low‐ and middle‐ versus high‐income countries as subgroup for our QES, we examined included studies for inclusion of qualitative evidence on these relevant groups.	

Abbreviations: LHW, lay health worker; QES, qualitative evidence synthesis.

### 1.2 How the intervention is conceptualised

In this QES, we focused on breastfeeding support programmes and the factors that influence women's engagement with them. We defined engagement as a context‐dependent, psychological state characterised by fluctuating intensity levels (Brodie et al., 2011). It occurs within dynamic, iterative engagement processes but does not equal involvement or participation only. Instead, it comprises cognitive, emotional, and behavioural dimensions. One of the core characteristics we emphasise is the necessity of a process of relational exchange to allow any engagement to occur, hence the focus on interactive components of support rather than logistics and material support (Brodie et al., 2011). We were particularly interested in factors that facilitate or constrain women's engagement with the programmes, both in terms of how breastfeeding support is perceived and experienced by them. In our synthesis, the target group was ‘mothers’ and ‘mothers‐to‐be’ (those who are pregnant but have not previously delivered a child). By using the term ‘women’, we refer to both.

We defined breastfeeding support primarily in terms of human actions, interactions or relational exchange efforts provided, both within specific programmes or on support by care providers such as a midwife or health visitor. We based our definition of support on the comprehensive definitions provided by McFadden et al. (2017) and Lumbiganon et al. (2016):

‘*Support could include reassurance, praise, information, and the opportunity to discuss and to respond to a woman's questions, and it could also include staff training to improve the supportive care given to women. It could be formally offered by universal or primary care providers in hospital setting or informally by lay people, trained or untrained, in a community or home setting. It could be offered to groups of women or one‐to‐one, including mother‐to‐mother support, and it could be offered proactively by contacting women directly, or reactively, by waiting for women to get in touch. It could be provided face‐to‐face or over the phone, and it could involve only one contact or regular, ongoing contact over several months* (McFadden et al., 2017)*. Support interventions could be on an individual or group basis, could include home visiting programmes, peer education programmes, or clinic appointments specifically aimed at imparting knowledge about breastfeeding and could involve prospective fathers or not. Interventions could also include an educational component, such as education session, printed information, video, peer counselling, and lactation consultation*’*. *(Lumbiganon et al., 2016).

### 1.3 Aims and objectives

The overall aim is to provide suggestions on how to improve breastfeeding support programmes to increase the level of satisfaction of women with support and to better match the expectations and needs of women.

The review questions are as follows:

1. How do women perceive or experience breastfeeding support?
  a. Which contextual factors influence women's overall engagement and responsiveness to breastfeeding support services or programmes?
  b. Which contextual factors influence women's satisfaction with breastfeeding support services or programmes?
  c. Which barriers or facilitators may have an impact on the choice of women to engage with or take part in breastfeeding support?
2. What are potential matches and mismatches that can be identified between women's needs and the way breastfeeding support services or programmes are currently rolled out?
  a. What do women consider the right moment to initiate support (antenatal vs. postnatal)?
  b. How long is support needed as perceived by women?
  c. What type of support or which components are appreciated or lacking?
3. What other benefits do women get out of support programmes apart from breastfeeding initiation and duration?

The specific objectives of the review are:

1. to identify and synthesise qualitative studies exploring factors that facilitate or constrain the engagement and satisfaction with breastfeeding support in two phases of the support process:
  a. breastfeeding support to initiate breastfeeding (antenatal phase)
  b. breastfeeding support to continue breastfeeding (postnatal phase)
2. to identify characteristics of the population, intervention or outcome that may be important to consider (e.g., relevant subgroups, outcome measures, questions that need to be considered).

## 2 METHODS

### 2.1 Eligibility criteria

We included studies that focused on women who are about to receive support (initiation), who currently receive support (continuation), or who have received support. We considered information on both perceptions (e.g., on the kind of support they wish to receive) and experiences of women. We targeted women who responded to the support and those who were motivated to enrol in such programmes, for instance, those approached with information before having delivered a child (mothers‐to‐be). The type of evidence collected in our synthesis also included participants’ satisfaction with components of a support programme provided, for instance, the quality of the support delivered by others, for example in terms of level of training, demographic and professional characteristics of the providers. No restrictions were placed on age, social status, ethnic background, or country of recruitment. This review has a clear focus on first‐hand accounts; hence we excluded the perceptions of women as reported second‐hand by their partners, family members, or health professionals.

The eligibility criteria are outlined in Table 2.

**Table 2:** Eligibility criteria

	Inclusion criteria	Exclusion criteria
**1 Types of studies**	– Grounded theory, phenomenological, narrative, action research, case, and visual studies – Qualitative methods for data collection, as focus groups, face‐to‐face interviews, observations, arts‐based methods or document analysis, and data analysis such as content analysis, thematic analysis, constant comparison analysis, or other qualitatively inspired analytical approaches – Mixed methods studies were included if qualitative data could be extracted separately	– Editorials, commentaries, opinion papers, conference contributions – Studies that did not provide a transparent audit trail of the methods used
**2 Topic of interest**	– Focus on women who are about to receive breastfeeding support (initiation), who currently receive support (continuation) or who have received support – The kind of support they wish to receive) – Experiences of women	– Studies that focus on the experience with breastfeeding – Studies on more general barriers and facilitators of breastfeeding
**3 Participants**	– Women who are women and mothers‐to‐be – Women who responded to the support programmes and those who were motivated to enrol in such programmes, for instance, those approached with information before having delivered a child (mothers‐to‐be) – No restrictions on age, social status, ethnic background or country of recruitment	– Perceptions of women as reported second‐hand by health professionals or significant others
**3 Settings**	– Health facilities, home‐based interventions (e.g., delivered reading materials; either online or in print), local support communities, and home support programmes (e.g., home visits)	none
**4 Types of interventions**	– Studies that focus on breastfeeding support programmes, including those exploring attitudes and views of women and those reporting on experiences of breastfeeding support – Interventions directly addressed to women	– Support programmes that only provided logistics (e.g., a room or a fridge) – Interventions at a policy level or those primarily aimed at health professionals
**5 Phenomena of interest**	– Women's (non‐)engagement with breastfeeding support programmes – Women's satisfaction and responsiveness to breastfeeding support programmes – Women's beliefs, attitudes, perceptions and experiences	

### 2.2 Search strategy

We systematically searched the databases CINAHL, MEDLINE, EMBASE, PsycINFO and Embase using keywords including breastfeeding, support, specific qualitative terms as well as experience terms. A combination of index terms and free‐text words were used (Heyvaert et al., 2016). Supporting Information S2 contains the MEDLINE search strategy. The search strategy was not limited by time, language, or publication type. Where and when relevant, we conducted ‘related article’ searches in the databases that offer such an option. We hand searched relevant journals and contacted authors of relevant studies and content experts for any unpublished work (Heyvaert et al., 2016).

### 2.3 Screening of studies

Screening of titles and abstracts was undertaken in Covidence. All titles and abstracts were screened in dual mode and were independently assessed for eligibility. Full‐text studies were screened in EPPI‐reviewer. EPPI‐reviewer has been developed by EPPI‐centre at the Social Science Research Unit in the Department of Social Science, University College London. While systematic reviews were excluded, their reference list was searched for relevant studies for inclusion. Disagreements at all stages were resolved through discussions and if required by involving a third reviewer. Supporting Information S3 includes a list of excluded studies and the main reasons for exclusion.

### 2.4 Sampling of studies

Breastfeeding support varies in nature. Support (programmes) may: (a) use multiple types of implementers; (b) diverse forms of communication; (c) consists of various components; (d) use different time points at which the intervention is initiated; and (e) target different groups within the overall population of women (Table 3). We took these dimensions into account when we described the characteristics of the included studies (Supporting Information: S1). We also accounted for these variations in our sampling approach in the form of a maximum variation strategy to ensure that the phenomenon was looked at from various points of view.

**Table 3:** Types of breastfeeding support programmes

Dimension	Variation	Supporting reference(s)
**(a)** Type of implementers of programme	– One‐to‐one support from health professionals (midwives, family physicians, nurses, International Board‐Certified Lactation Consultants, etc.) – Peer group support (drop‐ins, cafes, centres) – Support that is targeted at the core‐family (support for partners, etc.) – Support with no human involvement (books, helplines, websites, leaflets)	Abbass‐Dick et al. (2015), Hoddinott (2006), Kronborg et al. (2008)
**(b)** Form of communication	– Verbal communication – Written communication – Oral communication (podcasts, etc.) – Visual communication (animation videos, etc.) – Electronic communication (mobile phone text messages, apps, internet, etc.)	D'Auria (2011), Thomas and Shaikh (2012)
**(c)** Type of component added to the programme	– Educational sessions – Some sort of information provision – Assessment – Supervision – Measures that target the direct relation between mother and baby (breastfeeding immediately after birth, rooming‐in, etc.) – Interventions in case of urgent medical issues (mastitis, etc.) – Advocacy – Encouragement	Beake et al. (2012), Guise et al. (2003), Jaafar, Ho, Jahanfar, et al. (2016), Jaafar, Ho, and Lee (2016), Lynch (2010), Santos et al. (2015)
**(d)** Time point/period the programme is initiated	– Before conception and early pregnancy – During pregnancy – Immediately after birth – During the first months after birth	Fallon et al. (2016), Hannula et al. (2008)
**(e)** Persons targeted	– Women – Mothers‐to‐be – First‐time mothers – Socially disadvantaged women (e.g., low‐income) – Women with specific needs	Abdulwadud and Snow (2012), Bonet et al. (2015), Demirtas (2012), Gorman et al. (2009), Heck et al. (2006), Khoury et al. (2005), Kronborg et al. (2015), Lawrence and Lawrence (2005)

**Figure 1:**
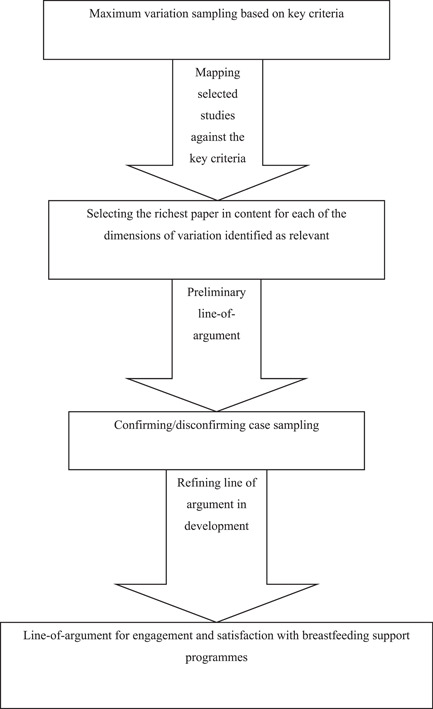
Sampling strategy. Based on Benoot et al. (2016).

In our QES we aimed for variation in concepts rather than an exhaustive sample, as large amounts of study data can impair the depth and therefore the quality of the analysis (Booth, 2011). For this QES, we combined multiple purposeful sampling techniques (Benoot et al., 2016; Figure 1). Our purposeful sampling framework was driven by a maximum variation sample logic (Suri, 2011). First, we decided on six key sampling criteria (types of implementers, types of components added to the programme, form of communication, timepoint of programme, persons targeted and setting) that would allow us to look at the phenomenon from multiple perspectives and transferred these criteria into a grid. Second, we sampled the selected studies that matched our eligibility criteria against the cells (representing variations of key criteria) of our grid. Based on our maximum variation sample logic, we selected at least one study for each cell in the grid, thereby securing that all relevant variations of the key sampling criteria were covered (e.g., written, verbal or haptic as variations of the key criteria form of communication). If there was a choice of studies, we chose those we judged as richer in content on both a descriptive and conceptual level. After applying this sampling strategy, we selected 14 studies for data extraction. Third, we constructed a preliminary line of argument, which we further refined by applying the fourth step; the inclusion of potential disconfirming cases that could challenge the line of argument we were building (Booth et al., 2013). This was done by selecting those with potentially contrasting or contradictory interpretations of collected evidence so far. An additional sample of 8 studies was selected as deviant cases resulting in a total of 22 studies. Their findings are the basis for the findings reported here. For an overview of the studies that were not sampled, see Supporting Information: S3.

### 2.5 Data extraction, analysis and synthesis

We performed data extraction in EPPI‐reviewer using a specifically developed form that we used to extract key themes and categories relevant to the review objectives (Supporting Information: S4). Categories included the content of information on support programmes, women's views and experiences of the support programme and the extent of its influence on their engagement with support programmes. We also extracted information about language, context, theoretical or conceptual frameworks and research methods. One reviewer extracted data from all included articles and a second reviewer double‐checked it.

We then used a thematic synthesis approach for data analysis (Thomas & Harden, 2008). Our analysis was built on the following stages:

1. *Familiarisation*: immersion in the content of included studies with the aims and objectives of the review.
2. *Identifying a pre‐defined coding structure*: Cargo et al. (2015) provided us with a comprehensive list of possible contextual factors that could influence intervention implementation or engagement with the intervention and thereby guided our analysis.
3. *Jotting*: we moved between the data and the themes covered in the coding structure. We also searched for additional themes until all the studies had been reviewed.
4. *Coding*: texts were coded according to meaning and content. We then developed descriptive themes and translated the concepts from one study to another and a hierarchical structure was created by grouping the codes based on similarities and differences between the codes. Analytical themes that go beyond the content of the original articles were then developed and reviewed independently to consider implications.
5. *Focus on equity*: we paid particular attention to possible differences in perceptions and experiences within and across settings (e.g., HIC and LMIC) as well as between groups of women (e.g., minorities as teenage mothers) when we wrote up our findings.

### 2.6 Assessing confidence in the review findings

Three reviewers (Hui‐Lin Cheng, Shoba Dawson and Theresa Bengough) used the GRADE‐CERQual approach to assess confidence in each finding (Lewin et al., 2018). After assessing each of the four components independently, we discussed each reviewer's decision. We discussed whether issues were critical or not and then reached consensus on minor, moderate or major methodological considerations. For example, the majority of the studies did not report any reflexivity and several didn't discuss why the choice of data collection/analysis was appropriate. This was judged as a major methodological consideration. Other reasons for downgrading for methodological limitations were poor reporting of all (or at least most of) criteria from half of the contributing studies. We typically downgraded a finding for concerns about coherence when some of the data from the included studies contradicted the review finding or when the underlying data did not explicitly support the review finding. We downgraded findings because of concerns about relevance in cases where the studies were from a narrow range of settings (e.g., only LMIC) or when only one group of implementers were included (e.g., only peer supporters). Downgrading due to data adequacy occurred when we had concerns about the quantity of the data supporting a review finding or when contributing studies were thin (Lewin et al., 2018).

As in all assessment exercises that impose an arbitrary, numerical cut‐off point to decide on quality levels, this was a subjective conclusion reached between authors. We present summaries of the findings and our assessments of confidence in these findings in Supporting Information: S5.

## 3 RESULTS

### 3.1 Description of the studies

**Figure 2:**
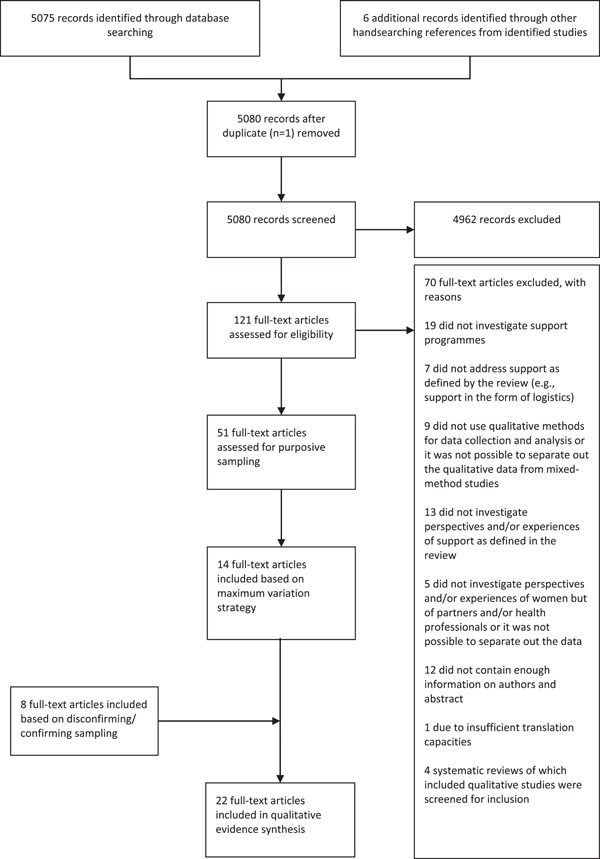
PRISMA flow chart

We identified 5075 titles and abstracts published on or before 30 November 2017. We considered 121 full‐text papers for inclusion in this synthesis (Figure 2). We identified 51 studies that met our inclusion criteria and 14 were purposively sampled. An additional sample of 8 studies was selected as deviant cases resulting in a total of 22 studies sampled for inclusion in the synthesis (Figure 2). Most of the studies used interview and/or focus group discussions as data collection method and only a few used other methods of qualitative data collection such as participant observation or written journal entries with answers to qualitative questions. One study used a think‐aloud method when using a pictorial representation.

### 3.2 Participants and settings

In all sampled studies, authors sought the perspectives of women participants. All studies included diverse women in terms of and not limited to ethnicity, socioeconomic backgrounds, level of education, age, language and parity, with most studies addressing explicitly but not exclusively first‐time mothers (*N* = 13). Of the 22 studies, 3 reported research in LMICs: South Africa (*N* = 1), Brazil (*N* = 1), Malawi (*N* = 1), 19 took place in HICs: the United Kingdom (*N* = 6), Sweden (*N* = 2), the United States (*N* = 7), Australia (*N* = 3) and Ireland (*N* = 1). These assignments are based on the World Bank's classification of income level.^1^ Eight studies focused on support programmes in a hospital or health care setting. Eight studies focused on support programmes in more than one setting. Of these, six studies focused on routine support from the hospital staff rather than a specific support programme. Two studies focused exclusively on support implemented in the home setting. One study focused on support in a group setting and one study focused on a virtual setting. Two studies did not report the setting.

### 3.3 Methodological quality of the studies

We appraised the quality of 51 studies that met the inclusion criteria using the CASP tool (Critical Appraisal Skills Programme [CASP], 2014). For each paper with primary data, two reviewers independently applied CASP. The first author screened the whole set and met separately with the co‐reviewers to discuss discrepancies. During the meetings with the project team only the papers with discrepancies were further discussed to reach consensus. All studies gave at least a brief description of the participants, sampling, methods, and analysis. Studies varied in the level of detail provided, but overall, there was poor reporting of context, sampling frames and research methods which made assessment difficult. Traces of researcher reflexivity were scarce in most studies (Table 4). It is of importance to mention that reporting does not necessarily equal quality. Poor reporting does not allow us to judge whether a study is trustworthy. We therefore did not exclude studies based on our assessment of methodological limitations but used these to assess our confidence in the findings.

**Table 4:** Methodological limitations of included studies based on modified Critical Appraisal Skills Programme (CASP) tool

Study ID	Was the context described?		Was the sampling strategy appropriate and described?		Was the data collection strategy appropriate and described?		Was the data analysis appropriate and described?		Were the findings supported by evidence?		Is there evidence of researcher reflexivity?		Have ethical issues been taken into consideration?	Overall assessment of methodological limitations	
Ahluwalia (2000)	Yes		Yes		Partial		No		Yes		Partial		No	Moderate to major	
Andreson (2013)	Partial		Partial		Partial		Partial		No		No		Partial	Major	
Andaya (2012)	Partial		Yes		Yes		Yes		Yes		No		Partial	Moderate to major	
Backstrom (2010)	Yes		Yes		Partial		Partial		Yes		No		Partial	Minor to moderate	
Bailey (2010)	No		Yes		Partial		Yes		Yes		No		Yes	Moderate	
Barona‐Vilar (2009)	Partial		Partial		Partial		Partial		Partial		No		Partial	Major	
Battersby (2002)	Yes		No		Partial		No		Partial		No		Partial	Major	
Beake (2005a)	Yes		Yes		Yes		Yes		Yes		No		Yes	Minor	
Beake (2005b)	Yes		Yes		Partial		Yes		Yes		Partial		Partial	Minor	
Beake (2010)	Partial		No		Yes		Yes		Yes		Partial		Yes	Minor	
Breedlove (2005)	Yes		Yes		Yes		Yes		Yes		Partial		Unclear	Minor	
Bridges (2016)	Yes		Yes		Yes		Yes		Yes		Yes		Yes	Minor	
Bula (2015)	Yes		Yes		Yes		Yes		Yes		Yes		Yes	Minor	
Burns (2017)	Yes		Yes		Yes		Yes		Yes		Partial		Partial	Minor	
Chaput (2015)	Partial		Partial		Partial		Partial		No		Yes		Partial	Moderate	
Condon (2012)	Partial		Partial		Partial		Partial		Yes		No		Partial	Moderate to major	
Condon (2015)	Partial		Yes		Yes		Partial		Yes		Yes		Yes	Minor	
Coreil (1995)	Partial		No		Yes		Partial		Yes		No		No	Major	
Craig (2010)	No		No		Yes		Unclear		No		Partial		Partial	Major	
Cripe (2010)	Yes		Partial		Partial		Yes		Yes		Yes		Partial	Minor to moderate	
Cross‐Barnet (2012)	Partial		Partial		Partial		No		Partial		No		Yes	Major	
da Rocha (2013)	Partial		Partial		Partial		Unclear		No		No		Yes	Major	
Engstrom (2000)	Partial		Yes		Partial		Yes		Partial		Partial		Partial	Minor	
Entwistle (2010)	Yes		Partial		Yes		Partial		Partial		Yes		Partial	Minor to moderate	
Fox (2015)	Partial		Partial		Partial		Yes		Partial		Partial		Partial	Moderate	
Gill (2001)	No		No		Yes		Yes		Yes		Partial		Yes	Moderate to major	
Hailes (2000)	Partial		Partial		Unclear		No		Partial		No		Partial	Major	
Hall (2014)	Partial		Partial		Partial		Partial		Yes		Partial		Partial	Minor to moderate	
Hoddinott (2006)	Partial		Partial		Yes		Yes		Yes		Partial		Partial	Minor	
Hong (2003)	Partial		Partial		Partial		Partial		Yes		Partial		Partial	Minor to moderate	
Hunt 2017	Yes		Partial		Partial		Partial		Partial		Yes		Yes	Minor to moderate	
Hunter 2015	Yes		Partial		Yes		Yes		Yes		Partial		Yes	Minor	
Ingram (2013)	Yes		Partial		Yes		Partial		Partial		No		Partial	Moderate	
Islam (2016)	Partial		Yes		No		No		Partial		Partial		Yes	Major	
Johnson (2016)	Yes		Yes		Yes		Yes		Yes		Partial		Partial	Minor	
Leahy‐Warren (2017)	Yes		Partial		Partial		Partial		Yes		No		Partial	Moderate	
Locklin (1994)	Partial		Partial		Partial		Yes		Yes		Partial		Yes	Moderate to major	
MacVicar (2017)	Yes		Yes		Yes		Yes		Yes		Unclear		Yes	Minor	
McFadden (2013)	Partial		Partial		Yes		Yes		Yes		Partial		Yes	Minor	
Meier (2007)	Yes		Partial		Yes		Partial		Partial		Partial		Partial	Moderate	
Muller (2009)	Partial		YES		Partial		Partial		Partial		No		Yes	Moderate	
Nankunda (2010)	Yes		Partial		Partial		Partial		Partial		No		Partial	Moderate	
Noble‐Carr (2012)	Yes		Partial		Yes		Partial		Yes		Partial		Yes	Minor to moderate	
Rossman (2010)	Yes		Yes		Yes		Yes		Yes		Yes		Yes	Minor	
Sheehan (2009)	Partial		Yes		Partial		Yes		Yes		Partial		Yes	Minor to moderate	
Thomson (2012)	Yes		Yes		Yes		Partial		Yes		Partial		Yes	Minor	
Thomson (2013)	Yes		Yes		Yes		Yes		Yes		No		Yes	Minor	
Thorstensson (2016)	Yes		Yes		Yes		Partial		Partial		Partial		Partial	Minor	
Wade (2009)	Partial		Partial		Partial		Yes		Partial		Partial		Partial	Moderate	
Weimers (2006)	Yes		Yes		Yes		Yes		Yes		Partial		Yes	Minor	
Whelan (2014)	Partial		Partial		Yes		Partial		Yes		Partial		Partial	Minor to moderate	

### 3.4 Findings

Four overarching process factors affecting the engagement with breastfeeding support programmes emerged from the thematic analysis: (1) information provision; (2) type of implementers; (3) delivery modes and (4) maternal care pathways. Each overarching category contains sub‐categories, the latter incorporating several findings. For each finding, we compare what women experience as constraints or nonoptimal promotion practice with their expectations of what adequate support should look like.

In the sections below, we report each finding and provide a link to the CERQual evidence profile table (Supporting Information: S5, tables 5–16). Additionally, we provide a table presenting supportive quotes at the end of each overarching process factor.

#### 3.4.1 Process factor: Information provision—The what

##### Elements that should be included in breastfeeding information (Supporting Information: S5, table 5)

1. Finding 1: Women do not want technical breastfeeding information (Supporting Information: S5, table 6)

Women perceived breastfeeding as a natural event discussed within a medical context by health professionals (Craig, 2010; Cripe, 2010; Rossman, 2010; Thomson, 2012). Women found medicalization of breastfeeding disturbing and did not support the use of technical and clinical language (Cripe, 2010, Thomson, 2012) as they found it inaccessible. Our findings highlighted that the language used by support providers can substantially impact on women's responsiveness to support programmes and generally on women's feeding decisions. Women expressed a need for language that is easily understood so that it enhances their knowledge and understanding (Thomson, 2012; Table 5, finding 1).

2. Finding 2: Women want consistent messages about infant feeding (Supporting Information: S5, table 6)

**Table 5:** Supportive quotes

Reference	Supportive quote	Study
Finding 1	*You could ask her (breastfeeding buddy) questions and she'd explain them in a fashion that you could understand without being too medical*.	Thomson 2012
Finding 2.1	*I laid there for hours and I called somebody because I said that he just hasn't fed and the baby just slept and his head looked very, you know what they're like after forceps… and they just sort of said “oh no, he'll feed when he's ready”…. if I had known then that's them stimulating your supply, I'll be a lot more confident next time, but in the end this sort of carried out sort of every hour and I was thinking “he must be starving and he is not getting anything”, and in the end the midwife said “oh leave him with us, we'll just give him a bottle” and I was so sort of shattered that I did, but I regret that now*.	Beake 2005
Finding 2.2	*To start off with it was a bit of nightmare because of conflicting advice from various people. The best advice I got from one of the previous nurse, then after that everything was conflicting. So that was hard*.	Islam 2016
Finding 2.3	*In the hospital they kept repeating that it shouldn't be painful, if you are doing it right it shouldn't hurt. And that wasn't particularly helpful because it was painful for me*.	Fox 2015
Finding 2.4	*We also get the same messages from MaiMwana counsellors who visit us in our respective homes. They tell us to start giving our children food like porridge and water after 6 months. We are also given the same advice when at the hospital soon after delivery*.	Bula 2015
Finding 3.1	*If I knew what was to come or what I could expect to happen then I wouldn't have freaked out so much. Because even with all the information that I had I was very, very unprepared, I wasn't prepared for the pain, or the bleeding nipples, or the cluster feeding. I didn't know that babies could feed for 6 h. I just didn't know it was possible*.	Fox 2015
Finding 3.2	*I knew a lot about breastfeeding, but once things started going wrong, I felt kind of like maybe I wasn't so prepared*.	Meier 2007
Finding 3.3	*Knowing what is in for them (the babies) would be important*.	*Noble‐Carr 2012*
Finding 3.4	*I would never have started. I'm serious because it's not that they push you to do it but it's like they give you an enthusiasm about doing it. It's like this is for your baby. Once you see your baby and you 're not able to bring your baby home and then you hear that your milk is the best milk for your baby ‐ it's like I've got to do this. My milk is what's best for my child*.	Rossman 2010
Finding 3.5	*For us who are HIV‐positive we should only be encouraged to breastfeed exclusively for 6 months not more than that because the child can easily get infected with the HIV virus that we have. It is painful to breastfeed knowing that your baby might get infected*.	Bula 2015

Women received inconsistent advice and information about breastfeeding (Ahluwalia, 2000; Beake, 2005; Bula, 2015; Condon, 2012; Cripe, 2010; Cross‐Barnet, 2012; Engstrom, 2000; Fox, 2015; Hong, 2003; Islam, 2016; Meier, 2007; Noble‐Carr, 2012). This is an individual as well as an institutional problem (within and across institutions). Nonoptimal communication practices at the individual level were experienced as health professionals contradicted each other and provided different recommendations about initiation and latching techniques, optimal duration of breastfeeding and when to introduce solid foods (Ahluwalia, 2000; Beake, 2005; Condon, 2012; Cripe, 2010; Cross‐Barnet, 2012; Engstrom, 2000; Hong, 2003; Table 5, finding 2.1).

Nonoptimal communication practices at an institutional level were experienced when women received different recommendations from staff within one institution or when they found different organisations promoting different feeding messages (Bridges, 2016; Cripe, 2010; Engstrom, 2000; Islam, 2016; Table 5, finding 2.2).

Inconsistent messages were also noted when breastfeeding flyers were distributed containing information on food that women were told not to eat. Consequently, women had to find information themselves (Bridges, 2016) and felt uncertain whether they were doing it right (Beake, 2005; Fox, 2015). On the individual level, women did not differentiate between health professionals and lay/peer supporters when they expect consistent information. If consistent information is given, women felt supported in their decisions. When materials were distributed, they felt it was important that content was consistent with what health professionals and lay/peer supporter's advised (Table 5, findings 2.3 and 2.4).

No differences regarding the type of women (age groups) or the type of setting (low‐ versus middle‐ or high‐income setting) were observed.

3. Finding 3: Women want realistic information on benefits as well as risks and challenges of breastfeeding (Supporting Information: S5, table 6)

Women considered breastfeeding information as idealistic rather than realistic, especially when they participated in antenatal preparation for breastfeeding (Battersby, 2002; Breedlove, 2005; Condon, 2012; Fox, 2015; Meier, 2007; Noble‐Carr, 2012; Thomson, 2012). Idealistic information (e.g., breastfeeding is easy and instinctive) was described as a nonoptimal communication practice that does not address predicaments or risks and thus hinders women from being prepared for obstacles (e.g., latching issues, mastitis and clustering). As a result, women were shocked when they experienced unexpected and unexplained situations (Breedlove, 2005; Fox, 2015; Thomson, 2012; Table 5, finding 3.1).

A realistic initial assessment of the predicament or risks to breastfeeding depending on individual woman was reported as optimal communication practice because it helped to piece together the reality of their situation (Breedlove, 2005; Fox, 2015; Meier, 2007; Thomson, 2012; Table 5, finding 3.2). Generally, women wanted information on the spectrum between exclusive formula and exclusive breastfeeding in the form of mixed feeding.

The need to know the risks of breastfeeding for the woman went hand in hand with the need for reiteration of the health benefits for children because it affirmed the decision for breastfeeding and reminded women why they continue (Bula, 2015; Da Rocha, 2013; Noble‐Carr, 2012; Rossman, 2010). Information about the risks of breastfeeding as opposed to any risks related to not breastfeeding, supported women in making a balanced decision (Table 5, finding 3.3).

Our deviant case analysis revealed differences in influential factors depending on the groups of women: women with babies in an intensive care unit found it more encouraging if supporters (re)explained the many health benefits. Providing breastmilk for a very low birth weight (VLBW) infant is a complex process, wherein women encounter numerous barriers and challenges to breastfeeding. VLBW infants may be unable to feed at the breast due to immaturity or illness and therefore women must express breastmilk for weeks or months. Reiteration of benefits gave women the feeling they could do something to support their child growing (Rossman, 2010; Table 5, finding 3.4).

We found a deviant case for the above finding in a study involving women living with HIV. This study's finding suggests that fear either about their existing health or passing on the virus to their babies via breastmilk outweighed the many benefits of breastfeeding for their infants (Table 5, finding 3.5).

#### 3.4.2 Process factor: Type of implementers—The whom

##### (Un‐)Supportive characteristics of implementers of breastfeeding support (Supporting Information: S5, table 7)

4. Finding 4: Women prefer the support of an implementer who has gone through similar experiences in relation to breastfeeding (Supporting Information: S5, table 8)

Shared experience was highly valued by women and made them more receptive to information on breastfeeding (Cripe, 2010; Rossman, 2010; Thomson, 2012). In settings where the use of technology is a necessity (e.g., ICU), women felt encouraged to engage with breastfeeding support when they realised that the implementers of support shared their real‐life experience (Table 6, finding 4.1).

**Table 6:** Supportive quotes

Reference	Supportive quote	Study
Finding 4.1	*Oh, she (a member of the social group support) had the same problem, and this is what she did and this is what worked and this is what didn't work. "You know just pretty much actual real‐world experience. Rather than," Oh I read a book that says you should do this or this." Even reading books for me wasn't all that helpful. What helped me the most was talking to the people that had actually been there*.	Cripe 2010
Finding 4.2	*There are people that work with this programme that have actually been through it. After talking with the BPC (breastfeeding peer counsellor), hearing her story, and seeing pictures of her child "going through it," she expressed relief and that the BPC "got me into believing that I wasn't the only one who's gone through this*.	Rossman 2010
Finding 5.1	*I really think that a way to get more women to do it (breastfeed) would be to get someone in here that is not burnt out, someone that wants women to do it wholeheartedly and are not just reading from a piece of paper*.	Ahluwalia 2000
Finding 5.2	*I still was not confident to meet someone, who I already did not know. I think because you do not have a face*.	Islam 2016
Finding 5.3	*It was like, like she [another participant] said, you felt like you were being treated like dirt if you would even think about giving a bottle*.	Ahluwalia 2000
Finding 5.4	*“I was left completely alone. I am sure if I had called for help, I would have got it, but I didn't ever ask for any and therefore nobody came to see me which was fine*	Beake 2005
Finding 6.1	*It was more like one‐on‐one, and it was more in‐depth, because the doulas really got down to the nitty‐gritty. ‘Okay, this is going to happen, so you might as well deal with it*.	Breedlove 2005
Finding 6.2	*I need to touch base with a community of people who know that it is normal and really great for my children*.	Bridges 2016
Finding 6.3	*And it's your one little event where you get to go out and meet other moms that are alike. Because you want to talk about what is going on in your life and if your friends don't have babies you know they're not going to want to talk about breastfeeding, why is one of your boobs bigger than the other*.	Cripe 2010
Finding 6.4	*I've always got the help. Always. No matter how silly the question is, they've always got an answer. And it's nice because they do remember your name, they do remember your baby, and it just feels, it feels nice. I think the advice you get here is so much better than that you get from GP's or health visitors, who sometimes don't seem to know that much about breastfeeding. They are experts in their field and they are mums themselves, which is always, experience speaks volumes*.	Fox 2015
Finding 7.1	*Knowing that it was a Star Buddy that run the group…and knowing that they are there helped and encouraged me to go along more regularly*.	Thomson 2012
Finding 7.2	*It provides such a great resource from the variety of information posted, which I can choose to read or not. I also really love that the information ABA (Australian Breastfeeding Association) provides is evidence‐based. I am a scientist, so this is really important to me*.	Bridges 2016
Finding 7.3	*I am just wondering why ABA is just so against formula … it says in this pamphlet that there are risks involved in feeding your baby artificial baby milk … I really find that quite judgemental and offensive*.	Noble‐Carr 2012

Support by another woman with a similar experience encouraged women about their own child's prospects and supported them in processing information concerning breastfeeding initiation and continuation (Table 6, finding 4.2).

On the level of individual characteristics, this highlights the importance that support persons share personal challenges, successes, and unique support mechanisms with respect to initiate and continue breastfeeding from the perspective of an experienced woman.

5. Finding 5: Women experienced disconnected encounters with hospital staff (Supporting Information: S5, table 8)

When support was provided as a core element of the role of a care provider at an institutional level, women experienced difficulties in initiating and establishing breastfeeding due to disconnected encounters. Women reported that staff were unavailable after birth due to their heavy workloads and that staffing patterns were counterproductive to the development of relationships (see finding 13; Ahluwalia, 2000; Beake, 2005; Bula, 2015; Fox, 2015; Hong, 2003; Islam, 2016). Some women felt that staff were burnt‐out (Ahluwalia, 2000). Women reported that although staff offered to call them in case of any issues, no one was available when they called for support. These women said that hospital staff did not have the time to sit with them and that often they gave women a bottle instead (Condon, 2012; Fox, 2015; Table 6, finding 5.1).

Women stated that maternity ward staff lacked knowledge about any support programme or had no information about certain elements of the programme (e.g., concrete tasks of the supporters). Some women tended to refrain from the programmes running at the ward (e.g., peer support) rather than engaging with it due to this lack of information (Table 6, finding 5.2).

We found evidence in two studies that revealed that women reported that infant feeding messages by hospital staff were strongly biased towards breastfeeding. Women experienced being pressured to breastfeed (Ahluwalia, 2000; Condon, 2012; Table 6, finding 5.3).

Our disconfirming/confirming sample revealed a deviant finding. Based on one study (Beake, 2005), we found that some women understood and accepted that routine institutional practices hindered hospital staff from supporting them to breastfeed (Table 6, finding 5.4).

6. Finding 6: Women value one‐on‐one support in the form of (online) community‐based supporters (Supporting Information: S5, table 8)

We identified two major forms of community‐based support models: First, support by a doula was described culturally sensitive as a response to community sociodemographic barriers and orientated towards a positive future. Doulas used informal and plain language and were perceived as authentic (Battersby, 2002; Breedlove, 2005; Table 6, finding 6.1). Women appreciated that doulas supported them by providing resources to initiate and continue breastfeeding. Women valued extended relational caring from the doula starting in early pregnancy.

Second, facilitated group support, where women were addressed one‐on‐one within a bigger group, were found to be highly supportive. This group support also included asynchronous support provided through Facebook groups (Bridges, 2016). Women liked to engage because they felt a sense of normality there (e.g., by exposure to breastfeeding in a group; Bridges, 2016; Cripe, 2010; Fox, 2015; Leahy‐Warren, 2017), especially for women who did not like to breastfeed in front of (male) family members or in a public place (Condon, 2012; Cripe, 2010). Women reported that group support was meaningful as challenges that might not necessarily be severe enough to call a professional were still frustrating, and support groups served as a good source of suggestions and instrumental help for these less severe issues (Cripe, 2010; Fox, 2015).

Our findings highlight a variety of benefits women reported from engaging with group support programmes:

1. The group serves as a safe place to practice breastfeeding skills and talk openly about own and other people's embarrassment and emotional status (Fox, 2015).
2. The group provides a sense of community (Bridges, 2016; Cripe, 2010; Fox, 2015; Table 6, finding 6.2).
3. The group offers opportunity to create friendships or play‐dates (Cripe, 2010).
4. Group programmes boost confidence through social support combined with reassurance, emotional support and guidance from skilled experts when a group is led by a facilitator (Cripe, 2010; Fox, 2015; Leahy‐Warren, 2017).
5. Being surrounded by women with similar challenges provides a sense of normality which helps to make sense of lived experiences (e.g., with baby's eating habits; Bridges, 2016; Cripe, 2010; Table 6, finding 6.3).
6. Because of the group's frequency, there is an opportunity for relationship building which is highlighted by group members remembering each other's names (women and babies alike; Cripe, 2010, Fox, 2015; Table 6, finding 6.4).

Our analysis revealed one deviant finding regarding the perceived value of community support groups based on one study of our confirming/disconfirming case sample. Women stated that meeting in a group to breastfeed implied a sense of hiding breastfeeding from society (Leahy‐Warren, 2017). This speaks towards a mismatch of values of group support.

7. Finding 7: Women judge the quality of the information provided as high when delivered in the context of official breastfeeding support programmes (Supporting Information: S5, table 8)

Women wanted credible support programmes, whether individual or group‐based (Bridges, 2016; Thomson, 2012). They experienced an official mission statement displayed as encouraging as this underlines a programmes’ quality. Our synthesis showed that a programme of an official association or organisation prompted women to actively joining. Perceived quality may therefore lead to higher participation rates (Table 5, finding 7.1). Women expressed feeling assured because they knew that the national breastfeeding association was responsible for an online support programme (Table 5, finding 7.2).

Our analysis revealed conflicting evidence as one study concluded that women refrained from official support programmes if expensive books or memberships were promoted (Noble‐Carr, 2012). Women did not appreciate it when the association explicitly noted risks associated with bottle‐feeding, finding it judgemental (Table 5, finding 7.3).

Conversely, another study found that some women considered the advice given by health workers from the hospital as more reliable and likely to have substantial health impact while the quality of counselling provided by peer counsellors was considered as below standard and unhelpful, even though the programme was a registered project with a charitable trust (Bula, 2015).

#### 3.4.3 Process factor: (Non) Optimal delivery modes—The how

##### Being supported as an individual (Supporting Information: S5, table 9)

8. Finding 8: Women want implementers of support to respect their individual choice of whether and how to breastfeed (Supporting Information: S5, table 10)

Women reported that they were presented with dichotomous choices when making feeding decisions. They described discourses around infant feeding as ‘black or white’ only, ‘can or cannot’, ‘success or failure’ and ‘breastfeeding or bottle‐feeding’. This resulted in breastfeeding perceived as an all‐or‐nothing practice (Fox, 2015).

A feeding plan was reported to be useful by women as a mode of delivery of support. They found it useful to develop the feeding plan together with an implementer of support (Breedlove, 2005; Fox, 2015; Meier, 2007; Rossman, 2010; Thomson, 2012) as it helped them find confidence in their own abilities to achieve their feeding goals. Women wanted these plans to be realistic (see finding 3) based on their socio‐biographical context and that the plans are evaluated continuously for signs that alter or reinforce the selected goals (Table 7, finding 8.1). This finding focuses on personal choice as a modus of engagement. It can be situated within both the individual level and a woman's socio‐cultural context. On the individual level, women placed their decisions on whether to breastfeed in their personal biographical context and this shaped (future) feeding decisions (Engstrom, 2000; Fox, 2015; Thomson, 2012).

**Table 7:** Supportive quotes

Reference	Supportive quote	Study
Finding 8.1	*She gave me leaflets and she would phone me every other day to see how I was going. I was so frightened and worried about getting mastitis because that had always stopped me because I get really poorly with it. She would phone me to make sure that everything was all right and I was not in any pain or anything and if I was worried about anything she would come and see me to check it… because I was worried about a lot, so she came out a lot to see me. She was brilliant, can't praise her enough*.	Thomson 2012
Finding 8.2	*I was looking at 5 weeks max. I said well I'm not going to be doing it for long anyway and A (peer supporter) said but even if it's only for a week, 2 weeks, you're giving her the best*.	Battersby 2002
Finding 8.3	*Like a thorn in the side, though I have sort of accepted it verbally, but I can still feel that there is a thorn in the side. I know that my family think that I should breastfeed, they haven't said so, but I still feel what they think: you should breastfeed your baby*.	Engstrom 2000
Finding 8.4	*They [organisation] did do a section on breastfeeding and she was excellent, and (partner) came away from that completely sold on it. He doesn't usually bother reading things, but he absorbed all that and he came away saying it's so good for her… He was adamant that was what we were going to try and do*.	Thomson 2012
Finding 8.5	*I knew nothing until somebody phoned me (once the baby was born)*.	Islam 2016
Finding 9.1	*They were pushing and hurting my breasts and I was like “for the love of God, just stop!*	Noble‐Carr 2012
Finding 9.2	*It might be their way of doing it, to get you going with the breastfeeding… but, I do not like it, It might be difficult to do it in another way. I do not think it is funny when someone is pulling your breast, but you have to accept the situation*.	Weimers 2006

Women often experienced a lack of support in choosing what works best for them and did not want to be judged for their chosen method. If their individual choices were not considered adequate, women often avoided advice given by the support implementers (Andreson, 2013; Battersby, 2002; Condon, 2012; Cripe, 2010; Fox, 2015). Women who chose to breastfeed for a short duration (compared to the recommended duration), wanted equal support from the implementers for this decision as women who breastfeed longer (Table 7, finding 8.2).

Central to individual women's choices are family, social and cultural networks. The socio‐cultural context is central to their decision on whether to breastfeed and in the long‐term for how long to continue (Ahluwalia, 2000; Bula, 2015; Breedlove, 2005; Condon, 2012; Craig, 2010; Engstrom, 2000; Fox, 2015; Leahy‐Warren, 2017; Meier, 2007; Noble‐Carr, 2012; Thomson, 2012). This centrality was stated to be both, negative and positive (Table 7, finding 8.3).

Some women experienced high pressure from their socio‐cultural network and found that support programmes couldn't counterbalance this (Craig, 2010), while others reported that hospital staff successfully built upon existing support from their personal network (Fox, 2015; Thomson, 2012; Table 7, finding 8.4). Generally, support programmes were often introduced only after birth. If this was the case, women preferred to be informed via phone soon after birth about available support options (Islam, 2016; Table 7, finding 8.5).

Our deviant case analysis revealed differences in feeding decisions influenced by women's culture in low versus high‐income countries. While synthesised findings from HICs point towards a stronger impact on the importance of feeding decisions on the individual level, findings from LMICs highlight a stronger impact on the socio‐cultural level. The socio‐cultural background of women impacted not only their feeding decision but also whether women were receptive to support programmes in general. In some cultures, being visited by a support worker was associated with certain medical conditions (e.g., HIV). As a result, women preferred travelling to a clinic or hospital, so their community was not aware of any support mechanisms (Bula, 2015).

Our deviant case analysis revealed differences on the type of women. Socio‐cultural networks had a strong impact on teenage‐mothers compared to other types of women. The findings highlight that teenage‐mothers experienced their peers having a negative perspective on breastfeeding and thus felt rather uncomfortable practising breastfeeding (Condon, 2012; Meier, 2007; Noble‐Carr, 2012). These findings highlight the need for sensitivity to the social norms of a specific group when support implementers work with teenage‐mothers.

9. Finding 9: Women do not like their breasts to be touched (Supporting Information: S5, table 10)

Women experienced haptic forms of breastfeeding support as unpleasant if these meant that their breasts were touched by someone, especially if unexpected. Women reported that this support was an insult to integrity, and experienced their breasts as objectified afterwards (Noble‐Carr, 2012, Weimers, 2006; Table 7, finding 9.1).

Although women seemed to accept this approach, the preferred alternatives, for example, demonstrating specific techniques with a model breast and doll (Weimers, 2006; Table 7, finding 9.2).

#### 3.4.4 Process factor: Care pathways—The where and when

##### Service designs (Supporting Information: S5, table 11)

10. Finding 10: Women want support to be easily and flexibly available (Supporting Information: S5, table 12)

Women found it difficult that many forms of support (e.g., home visits) needed to be planned and scheduled and could not be adapted easily to quickly changing life situations. Women appreciated immediacy and flexibility of support, especially if an issue was urgent (Bridges, 2016; Bula, 2015; Noble‐Carr, 2012; Thomson, 2012). Online groups or telephone helplines were reported to be flexible services that can be accessed anytime without being physically present (Bridge, 2016; Table 8, finding 10.1).

**Table 8:** Supportive quotes

Reference	Supportive quote	Study
Finding 10.1	*The times that I've needed support have been urgent and someone is always right there online with their experience to help you through*.	Bridges 2016
Finding 10.2	*It might be their way of doing it, to get you going with the breastfeeding… but, I do not like it, It might be difficult to do it in another way. I do not think it is funny when someone is pulling your breast, but you have to accept the situation*.	Battersby 2002
Finding 10.3	*I think she could have been around for five or six times, but she telephoned more often and I think perhaps the ringing was more important than the coming round to me, because she'd ring and say how is it going today and if it wasn't going very well she would come round but if it was going alright she didn't and that was fine by me*.	Thomson 2012
Finding 10.4	*The times that I've needed support have been urgent and someone is always right there online with their experience to help you through*.	Bridges 2016
Finding 10.5	*What I did beforehand was I rang the Breastfeeding Helpline and they were really, really good as well, and that's kind of what made me think you know what, there's different responses to how you feed your baby, conventional formula versus breastfed, and I thought I need to find a place to go to where I've got likeminded people and that grew my confidence to be honest as a new mum and as a breastfeeding mum*.	Fox 2015
Finding 11.1	*It is also important to be visited at home because you are relieved from the burden of going to the hospital every time because some stay very far from the hospital, and when they visit you at your home, you are relieved as well*.	Bula 2015
Finding 11.2	*There's a lot of people come in when you're in hospital and everything's thrown at you. But they came in and told me that they'd visit me at home as soon as I got home… asked me if everything was OK, talked me through any questions that I had, gave me a few leaflets on things I think …and then as soon as I got home they came, I think the first day once I got home they were here helping me*.	Thomson 2012
Finding 13.1	*Just to double check everything — even though he is feeding — just to make sure I've got it right — the main thing is the reassurance to know what I am doing is correct. I wish I could take a midwife home with me so she could help me put it in right every time it's just daunting — because I don't know if I'm doing it right or not and without their assistance and guidance*.	Craig 2010
Finding 13.2	*There's a lot of people come in when you're in hospital and everything's thrown at you. But they came in and told me that they'd visit me at home as soon as I got home… asked me if everything was OK, talked me through any questions that I had, gave me a few leaflets on things I think… and then as soon as I got home they came, I think the first day once I got home they were here helping me*.	Thomson 2012
Finding 14.1	*They stayed there with me the whole time when I was really frustrated, really worked with me. They were really helpful*.	Hong 2003
Finding 14.2	*He was just on my breast 24/7… and I couldn't do nothing what I wanted to do… so I went to my health visitor and I said, ‘What would you say was the best option?', because… I didn't want to, like, give up altogether, and she said to me to start trying to express so I tried to express it, I couldn't express… so then I started a bottle through the day and breastfeeding at night and then I stopped that… and just went straight to bottle because he was so hungry, I needed to put him up onto a hungry baby milk*.	Condon 2012
Finding 14.3	*My second nurse brought her in during the night and I didn't know how to [breastfeed] and the baby was crying. So, she just watched me struggle with it. I mean, she had no suggestions to help me. I'm a first‐time mom and she just kind of looked at me, like [she] expected me to know what I was doing*.	Hong 2003

Similarly, women experienced travelling elsewhere to engage with breastfeeding support as a constraint. Technology‐based services gave them a sense of control (Battersby, 2002; Beake, 2005; Bridges, 2016; Bula, 2015; Fox, 2015; Hong, 2003; Thomson, 2012; Table 8, findings 10.2 and 10.3).

Women who engaged with an online peer support group reported that it encouraged their face‐to‐face experience as other women talked about it in a positive way (Bridges, 2016; Table 8, finding 10.4).

This finding is supported by another study where women stated that they received information about a support group when they called a breastfeeding helpline (Fox, 2015; Table 8, finding 10.5).

11. Finding 11: Women perceive benefits of home visits in combining various forms of support (Supporting Information: S5, table 12)

Women often experienced unsupportive, unconnected encounters with health professionals (see finding 7). Women wanted support in a stress‐free environment where they could talk about sensitive issues (Bula, 2015; Rossman, 2010; Thomson, 2012). Support in the form of home visits in a woman's home offered them a protective atmosphere (Table 8, finding 11.1).

In addition, they valued the fact that home visits offer paired support. As an example, women reported it to be useful that the supporter brought a pump to their home, hence offering instrumental support and paired this with emotional support by reassuring and encouraging them in their endeavour (Rossman, 2010; Table 8, finding 11.2).

Our disconfirming/confirming sample revealed a deviant finding: in LMIC women often needed to travel to hospitals or clinics for required, life‐saving services that were not related to breastfeeding. These women showed a tendency to refrain from home visits, mostly because support persons could not offer other required services (such as immunisation for their children; Bula, 2015).

##### Care pathway timeline (Supporting Information: S5, table 13)

12. Finding 12: Women want information about breastfeeding support options in early pregnancy (Supporting Information: S5, table 14)

Our findings revealed that breastfeeding support programmes were inadequately promoted during early pregnancy but also postnatally. Women experienced a lack of knowledge about where to find and how to access support programmes (Craig, 2010; Fox, 2015; Hong, 2003; Islam, 2016; Noble‐Carr, 2012).

13. Finding 13: Women want continuity in breastfeeding support (Supporting Information: S5, table 14)

Throughout their maternity care, women experienced the involvement of several professionals. Involving several professionals in their care pathway increased the risk of receiving inconsistent messages concerning breastfeeding, which is a barrier for them to engage with breastfeeding support (see finding 2). Women want continuity of care in the form of emotional and physical support starting during pregnancy, labour, birth, and mothering from maternity wards to their home (Table 8, finding 13.1).

Women wished to build trust with people supporting them through pregnancy, labour, birth and mothering (Breedlove, 2005; Bula, 2015; Craig, 2010; Fox, 2015; Rossman, 2010), with some stating that a friendly or familial relationship is supportive (Battersby, 2002; Rossman, 2010). One study found that it was supportive that the midwife who ran the antenatal classes was also the midwife supporting them during birth and postnatally (Fox, 2015). Another study showed that relational care from a doula starting in early pregnancy to early mothering encouraged women to continue breastfeeding (Breedlove, 2005). Another study found that women who were only supported in the hospital setting reported that they felt dependent on the hospital staff whereas the perceived aim was to breastfeed independently after hospital discharge. This would be supported by additional home visits after hospital discharge as they provide reassurance (Fox, 2015; Thomson, 2012; Table 8, finding 13.2).

14. Finding 14: Women perceive the optimal duration of physical support as the observation of whole feeds (Supporting Information: S5, Table 14)

Women indicated that only the initiation of feeding is supported and observed by implementers of support. They reported that as soon as a baby was successfully attached to the breast, supporters tended to leave (Bula, 2015; Condon, 2012; Hong, 2003). Consequently, subsequent feeds were not supported, and women felt left alone. Our synthesis shows that this constraint concerned the institutional as well as the individual level in the form of one‐on‐one support. Women valued supporters who took time to sit with them and observed whole feeds as this allowed practical and/or technical support such as help with ‘positioning’ and ‘latching on’ (Table 8, finding 14.1).

If breastfeeding was going well, proactive support declined rapidly (Condon, 2012; Table 8, finding 14.2).

The additional analysis of a disconfirming/confirming sample revealed a deviant finding: one study found that women did not consider it useful that supporters sat with them during the whole feed (Hong, 2003; Table 8, finding 14.2).

## 4 DISCUSSION

In this review, we focused on breastfeeding support programmes and the factors that influence women's engagement with them. Consistent with Brodie et al. (2011), we defined engagement as a context‐dependent, psychological state characterised by fluctuating intensity levels. It comprises cognitive, emotional, and behavioural dimensions. Breastfeeding support is primarily defined in terms of human actions and interactions, or relational exchange efforts provided (i.e., support that goes beyond merely providing logistics to facilitate breastfeeding, such as providing a room or a fridge at the women's workplace; Fewtrell et al., 2020; McFadden et al., 2017).

In line with the insights from other reviews (Fraser et al., 2020; McInnes & Chambers, 2008), our findings highlight that information provision is often not in line with the needs and expectations of women or does not reach them. Therefore, breastfeeding promotion should be integrated antenatally into wider child and maternal health interventions to support initiation rates. Furthermore, our findings reveal that most information regarding breastfeeding support is too idealistic; this seems to increase the risk of feeling unprepared and overwhelmed by unexpected real‐life events (e.g., mastitis, painful nipples). Our findings further suggest that women generally dislike health service support on an institutional level. They mention issues such as time pressure due to lack of staff, unhelpful practices such as handing over a bottle instead of taking the time to help with latching techniques, as well as conflicting advice. Our findings further highlight that women experience general support as invasive rushed, with no time for follow‐up questions. There is a missed opportunity to inform women about additional support resources (Hogg, 2017).

It is important to consider women's socio‐biographical context in relation to breastfeeding as it not only shapes actual and future feeding decisions but also how women situate their decisions in their personal and social context. This appears to be relevant for one‐on‐one support. Peer support has also been highly valued by women in our QES due to various characteristics attributed to peer supporters, that breastfeeding women reported to be positive and helpful. These attributes comprised sharing real‐life experiences, being connected to a similar community, normalising breastfeeding challenges and having a safe place to practice skills and talk openly. We advise that these attributes should be considered for the recruitment of implementers of support for any breastfeeding support programmes. Our QES suggests that engagement with informal support interventions prompts women to refer to further support services if they are recommended by other peers.

Finally, our QES highlights that the optimal maternity care pathway is patient‐centred, covering the postnatal months for as long a mother wishes to breastfeed. Continuity of the same implementers of support is said to be beneficial and this is supported by other studies (Bjurling‐Sjöberg et al., 2015; McFadden et al., 2017). In line with previous studies, the findings suggest that women perceive a trusting relationship with their supporters as helpful in initiating and continuing breastfeeding (Crossland, 2019; Fraser et al., 2020; Miller, 2016; Redshaw, 2018). We identified a variety of reported advantages of informal support interventions in the form of one‐on‐one support (e.g., Doula) but also in group support (e.g., community Baby Café).

Our QES also highlights some unique findings compared to other reviews. First, it illustrates the importance of one‐to‐one support interventions that are reported to be useful because support persons tend to have more time to sit, talk and most importantly, observe whole feeds as compared to general or standard support. Second, it emphasises the differences in experiences of women living in different conditions and geographical settings. Most women in LMICs felt motivated to engage with programmes when an official and trusted organisation was behind the programme. They felt more comfortable engaging with a trained health care professional than with peer supporters even when they were backed up by an official organisation. They linked perceived quality of support to professional competencies and skills. This tension between peer supporters being reported to deliver lower quality or being less knowledgeable on the one hand and high appreciation of women for their availability on the other hand may be rooted in the distinction women make between informational and emotional support. Another reason for this might be related to the socio‐cultural background where collective belief patterns outweigh, and health workers were considered more competent and skilled due to their education and profession whereas peer counsellors are lay people without specific education. Third, it highlights that breastfeeding support for women with HIV differs from support delivered to other women. Mixed feeding is not recommended in this group due to a higher risk of transferring the virus to the baby (Kassa, 2018). It is therefore crucial that everyone from the familial network who is responsible for taking care of a baby is aware of medical guidelines on this matter and is involved in the support programme. It is important to acknowledge that there may be complexity if not everyone involved in care is aware of the infant's HIV status. Fourth, we also found that women tend to refrain from breastfeeding support in the form of home visits for the following reasons: women in low‐income settings are forced to travel to hospitals or clinics for required, life‐saving services that are not related to breastfeeding (e.g., immunisations for their children). In this case, women found home visits not necessary, because the support person could not offer these other required services. It may then be a pragmatic choice to keep only appointments at the clinic that seemed more relevant. Women were also fully aware of the risk for stigmatisation due to the hospital‐mediated care, as people in the community could draw the link between having HIV and visiting the clinics.

Independently from geographical settings and differences in the characteristics of women, our findings show that breastfeeding support is an individual rather than a normative need. Still, there is a tension here because what is best in theory for an individual does not always fit social norms. Most of the challenges women reported in our included studies were related to socio‐cultural context and collective belief patterns of the women's social networks, including their families. Our findings suggest that these impact extensively on engagement with breastfeeding support programmes and services which is in line with the literature (Amir et al., 2021; Kim et al., 2017; Leeming et al., 2022). Before a support programme is developed, conflicting norms and traditions about infant feeding between professionals and women need to be identified. Programme developers and service providers should develop culturally sensitive support programmes and implementers of support need to be sensitive to socio‐cultural and personal backgrounds of each woman they offer their services to (Fewtrell et al., 2020; Leeming et al., 2022).

Based on the overall body of evidence of our QES, we argue that the optimal maternal care pathway is shaped by both informal and formal forms of support. These types of support have different purposes, but their functions are complementary. Our QES suggests that formal support in the form of universal or health professionals providing care as core element of their professional role focuses on the technological level whereas informal support makes up for deficits on the emotional, relational level and supports the normalisation of real‐life experiences. Efforts have been made to explore informal or nonprofessional forms of support (Fraser et al., 2020; Glenton et al., 2013) and their effectiveness has been proven (Jolly et al., 2012; Buckland et al., 2020). In line with previous studies, our QES suggest that women favour informal support over formal support (Beake et al., 2012; McInnes & Chambers, 2008) and that informal support has the potential to make up for deficits on an institutional level as for instance, lack of time to develop a relationship‐based care (Glenton et al., 2013).

### 4.1 Methodological challenges

Some of the methodological challenges we encountered were related to the primary study level. We found considerable heterogeneity concerning components of support and overall, most studies did not give sufficient detail on the type of support, especially the specific components of the programmes. For example, educational and counselling components of programmes were used interchangeably without proper definition (e.g., a breastfeeding counsellor runs an antenatal education programme). This is in line with the conclusion of a recent review (McFadden et al., 2019) which found that most studies did not give sufficient detail to make a judgement, often presenting a combination of both. All included studies featured a diverse sample of women in terms of ethnicity, age, level of education, socioeconomic background, language, parity, and so on, but no study presented a subgroup analysis. Applying a narrower focus and stratified analysis on the primary level could have added important information about values and priorities within different types of women, as differences between the types of women could reveal additional barriers to programme success (e.g., twin mothers vs. women with one infant).

The methods used in our included pool of studies may potentially limit the applicability and completeness of our data. All studies made use of individual or group interviews and focus group discussions as their primary method of data collection. Very few studies used observational methods, diaries, online social media platforms or visual types of data collection. While interviews and focus groups allow researchers to collect data on what people say, observational methods would allow researchers to collect data on how people react to a specific intervention. Our findings suggest that women experienced the direct touch or manipulation of their breasts as an insult, intrusive and a barrier for breastfeeding support. The potential inclusion of studies featuring ethnographic, observational methods could have been appropriate for understanding this situation better.

## 5 CONCLUSION

To develop our implications for practice and policy, we applied the CONSENSYS approach (Bengough, 2021). CONSENSYS is an instrument that supports reviewers to flag out relevant contextual factors potentially influencing programme implementation in the form of questions.

### 5.1 Implications for practice

Supporting women to initiate and continue to breastfeed is a complex process. Practitioners and service developers need to be aware that throughout the whole continuum of maternity care, women's breastfeeding support needs are dynamic, and it is unpredictable and uncertain when challenges and needs arise. The emotional and physical difficulties sometimes associated with breastfeeding may require diverse forms of support, also in combination, to counteract challenges.

Women's need for support was very wide, and support needs did not differ between the type of women. Women have similar core issues; hence breastfeeding support should be understood as a universal public health intervention. They need support and accurate support is often lacking in their living environment. One of the reasons for this is a lack of workforce, both on an institutional and community level. The evidence highlights the importance that emotional and appraisal support, as well as a stress‐free environment, have for women. This environment is hard to create within the hospital sector, even more, if support is to be offered universally across private and public health care. The social environment and the community seem to offer that space with informal support mechanisms making up for deficits on the institutional level. Women assign distinct roles to each support provider within their maternity care. While to them, staff on an institutional level provide technical and informational support, peer supporters on the community level can be engaged with deeper, on a level of trust and friendly relationships. Mechanisms to build trust happen at an emotional layer in the form of reassurance, appraisal and counselling. Practitioners and service developers should therefore consider combining these two forms of support within a programme wherever possible.

The concept of ‘timing’ is dominant in the context of breastfeeding support. Different time points become relevant because breastfeeding women have short‐term (initiating breastfeeding, knowing where to turn to for support), midterm (e.g., latching difficulties, appraisal) and long‐term needs (e.g., continuation, breastfeeding in a work environment). Awareness for this dynamism and hence flexibility within a support programme is therefore crucial to successfully engage women. Women want to be prepared for breastfeeding, and they want to be prepared as early as possible. The best time point for this in the perception of women is early pregnancy. The ideal information is balanced between benefits and harms, or risks associated with breastfeeding for both women and babies. Reiteration of the benefits of breastfeeding can support women to overcome breastfeeding challenges. As these are unpredictable, programme developers may consider different ways of promoting breastfeeding and support programmes. We recommend engaging women or at least consumer networks in promotion strategies.

The following questions may help stakeholders assess whether planned breastfeeding support interventions adequately address the issues that are important to women (Bengough, 2021):

1. Do all women within a region have equal access to the intervention? Are there any barriers that should be tackled (e.g., social norms that do not make it possible for some women to engage with the intervention)?
2. Is there any stigma towards the support implementer (e.g., home support is linked to a woman having HIV)?
3. Can a feeding plan be developed with each woman, giving her autonomy over how to feed her infant?
4. Does the staffing pattern allow women to experience consistency and establish a trusting relationship with the support implementer?
5. Is funding secured to provide adequate staff that can spend sufficient time with women (e.g., to observe whole feeds)?
6. Is it possible to combine health care and peer support to secure follow‐up and continuity of care in the long term?
7. Is there a possibility to include a telephone helpline as an additional element to a programme to provide general information on where women can find support including support in acute cases (e.g., mastitis)?
8. Is information about breastfeeding support communicated to women during early pregnancy? Can partnerships be created that support this information dissemination (e.g., with gynaecologists, community midwives and influencers)?
9. Is information within breastfeeding support communicated to women in a clear and simple manner in diverse formats?
10. Do health care staff provide the same information as found in materials they disseminate? Do health care staff within an institution provide consistent information?
11. Are competencies acquired by specific education reported to be more important than peer support that is provided by an official organisation or NGO? If so, may it therefore be important to consider whether peer supporters can be provided with specific training modules that can help raise the quality of support perceived by programme recipients?
12. Do support implementers provide women with realistic information tailored to their individual, biographical situation (e.g., if a woman experienced painful latching with her first child, it is likely she will experience this again with her second child)?
13. Do implementers of support respectfully communication with women in an empathic, context‐sensitive, and non‐judgemental way? Are they open to various ways of infant feeding (e.g., mixed feeding)?
14. Are they sensitive to intrusive behaviour (unexpected touch of women's breasts)?
15. Are they sensitive to the fact that women's needs are dynamic and may change due to a variety of challenging circumstances and are hence flexible in their forms of support?

### 5.2 Implications for policy

It is important that policymakers consider breastfeeding support a public health issue. On a policy level awareness is needed on how to frame messages. Normalising could be the key to successfully engage with and keep women in breastfeeding support programmes. Any breastfeeding (support) promotion should be realistic and use plain language. Using and integrating real‐life experiences can contribute to this normalisation. It is important for policymakers to be aware that women may need support throughout various time points in their breastfeeding endeavour, most importantly that they can access information about where they can get support in case they struggle.

Awareness for the cultural and social context of groups of women is crucial to support each individual woman to breastfeed. In cultural contexts where education and training are reported to be more important than shared real‐life experiences, a focus may be made in the promotion on the competencies and skills the support implementer brings. To understand and increase cultural and societal awareness, collaborative efforts must be made to establish partnerships between health care institutions and official organisations, nongovernmental organisations and societal stakeholders. Informal support is valuable for women. Influencers may help disseminate institutional information and provide it within the public domain. Further, we identified gaps in existing care pathways: first, there is a need to embed peer support culture on an institutional level. Second, there is a need for more training in delivering support programmes adequately to the needs of women that are raised in this QES and training resources for both health care staff and peer supporters need to be made available to them.

The following dimensions with matching questions may help local policymakers to assess whether the breastfeeding support interventions they are planning adequately address women's needs:

1. Is funding secured to establish a national breastfeeding telephone helpline that identifies and communicates all regional and local support mechanisms to women?
2. Are there enough support implementers? If yes, is there a need to train an adequate number? If no, may it be an option to shift the focus on the core family or peer support?
3. Can digital platforms be used to promote the programme?
4. How should the message of the programme be publicly conveyed? Can a breastfeeding woman be portrayed in public?
5. Can programme recipients be involved in promotion strategies to support normalisation of the issues breastfeeding women face (e.g., using photographs of women)?
6. Are there any other relevant political stakeholders from other sectors (social or environmental) that could be involved in (a) funding and/or (b) promoting the programme and/or identifying local facilities where the programme can be implemented?
7. Are there any existing relevant programmes or implementers that could be invited into the intervention as a form of broader intervention logic (availability of local support)?

### 5.3 Implications for future research

Our QES highlighted several research gaps. First, there is a lack of research on programmes specifically designed for women with multiple births. None of the included primary studies explored perceptions and experiences of support programmes for this group of women. This is consistent with the finding of a review on the effectiveness of breastfeeding support for mothers with multiples of Whitford et al. (2017). Second, we found no evidence on support programmes that specifically target the core family to help us understand how to discourage cultural beliefs and habits that hinder women to engage with breastfeeding support or on the other hand assist them in doing so. This kind of evidence could be relevant, especially for LMICs where familial networks impact strongly on infant feeding decisions (Bula, 2015). Third, studies that focus on support programmes in women's homes are scarce. Breastfeeding support, both antenatal and postnatal, tends to happen mostly at an institutional level. Our findings suggest that women who are not enroled in the institutional health care system before birth have a higher chance of being excluded from support programmes.

In general, we believe that subgroup analyses based on setting and type of participant would help to identify underlying cultural or social factors (e.g., shared beliefs) that influence engagement with support programmes. More studies might be needed in low‐income settings but also in the home setting in both, LMICs and HICs.

More research is needed on women's preferences around the timing, amount, and content of breastfeeding information to help tailor promotion strategies. Research on how to integrate peer support into the institutional level would be beneficial particularly in relation to the initiation phase. However, in general, there is a lack of qualitative evidence on the continuation phase of breastfeeding. Comprehensive, longitudinal‐based support could have an even greater impact on breastfeeding initiation and continuation beyond the first postnatal month. It deserves more attention.

## AUTHOR CONTRIBUTIONS

Theresa Bengough and Karin Hannes conceptualised the review and prepared the protocol. Theresa Bengough led the review process and prepared the review with guidance and revisions from Karin Hannes. All authors actively participated in screening of titles and abstracts as well as obtaining data, data extraction. All authors also contributed to the interpretation of the evidence, development of the logic model, and editing of the review.

## CONFLICT OF INTEREST

The authors declare no conflict of interest.

## Supporting information

Supplementary information.

Supplementary information.

Supplementary information.

Supplementary information.

Supplementary information.

## Data Availability

The data that support the findings of this study are available from the corresponding author upon reasonable request.

## Footnotes

1 World Bank country classifications by income level: 2020–2021.
