# Supplementary material for: Factors that influence women's engagement with breastfeeding support: A qualitative evidence synthesis
Source: Matern Child Nutr. 2022 Aug 25;18(4):e13405. doi: 10.1111/mcn.13405 (PMC9480951; doi:10.1111/mcn.13405)
Supplement: Supplementary file 1 — Supplementary information. [file MCN-18-e13405-s002.docx]

## Characteristics and references of studies meeting eligibility

Supplementary 1 gives an overview of all studies that met our eligibility criteria. In the top row, we label each study as included (considered in our final pool of studies) or sampled (considered in evidence synthesis). A sampled study therefore relates to the maximum variation strategy.

**Ahluwalia 2000**

| Sampled |  |
| --- | --- |
| Context (Country and income level, Setting) | Georgia, USA  High Income country  Hospital and telephone based |
| Study aim | To evaluate the impact of breastfeeding promotion strategies on breastfeeding initiation among WIC participants in Georgia. |
| Participant information | Low-income women who were feeding their infants |
| Intervention delivery | Multiple intervention delivered in hospital setting or via telephone |
| Study design (data collection and analysis) | Mixed-methods study. Data from focus groups and two individual interviews conducted as a part of the evaluation study was used.  No information available on how data was analysed. |

**Andaya 2012**

| Included |  |
| --- | --- |
| Context (Country and income level, Setting) | New York, USA  Urban region; High Income country  Hospital |
| Study aim | This study as part of a larger RCT examined women’s perceptions and reported effects of routine, primary care-based interventions to increase breastfeeding. |
| Participant information | Multi-ethnic (White/non-Hispanic; Black/non-Hispanic; Hispanic; Asian/Paciﬁc Islander/other)  Low-to middle income  English- Spanish speaking  Multi parity |
| Intervention delivery | Multiple intervention in hospital setting |
| Study design (data collection and analysis) | Mixed-method, RCT with qualitative interviews. Telephone interviews.  Data was analysed using content analysis |

**Andreson 2013**

| Sampled |  |
| --- | --- |
| Context (Country and income level, Setting) | South Africa  Urban region; middle income country |
| Study aim | This pilot study explored the feasibility of implementing a feeding buddy system to provide a mother with support to achieve her infant feeding goal. |
| Participant information | Women with specific health conditions (HIV) enrolled in clinic-based PMTCT program |
| Intervention delivery | Multiple component intervention at hospital and home |
| Study design (data collection and analysis) | Face to face in-depth interviews.  Conventional content analysis resulting in a coding framework. |

**Backstrom 2010**

| Included |  |
| --- | --- |
| Context (Country and income level, Setting) | Sweden  County with Urban, rural and sub-urban regions; high income country  Community, hospital and home |
| Study aim | The aim of this study was to investigate women’s experiences and reflections of receiving breastfeeding support, and midwives’ experiences and reflections of giving breastfeeding support. This study is part of a larger intervention study that includes a process-oriented program on breastfeeding management and promotion for health professionals in prenatal and child health centres in the study area. It investigated whether a training intervention within the care team of antenatal and child health centres would improve maternal perception of support and strengthen maternal feelings for the baby. |
| Participant information | first time mothers  other: varied in regard to social background, birth outcomes, time for discharge  from hospital and different experiences of birth and support, in order to clarify women's views of breastfeeding support |
| Intervention delivery | No information available. Delivered at community, hospital and home. |
| Study design (data collection and analysis) | A qualitative design with face-to-face interviews.  Data was analysed using content analysis. |

**Bailey 2010**

| Included |  |
| --- | --- |
| Context (Country and income level, Setting) | UK  High income country  In hospital, home or both. |
| Study aim | To explore and clarify the views of first-time mothers by addressing the question: What level of support do first-time mothers want from health visitors during the postnatal period? |
| Participant information | First-time mothers (with babies aged 3-6 months) |
| Intervention delivery | Multiple interventions delivered either at home or in combination. |
| Study design (data collection and analysis) | Face to face interviews.  Data was analysed thematically. |

**Barona-Vilar 2009**

| Included |  |
| --- | --- |
| Context (Country and income level, Setting) | Valencia, Spain  Urban region; high income  Clinics and home |
| Study aim | The aim of this study was to explore women’s perceptions and personal experiences about the inﬂuence of formal and informal social support on the decision to breast feed, and also to breast-feeding initiation and duration. |
| Participant information | First-time mothers during pregnancy divided among three different focus groups stratified per socio-economic background and education  Multiparous women |
| Intervention delivery | Multiple interventions delivered in clinics and at home |
| Study design (data collection and analysis) | Focus-groups and individual in-depth interviews.  Grounded Theory approach |

**Battersby 2002**

| Sampled |  |
| --- | --- |
| Context (Country and income level, Setting) | North of Sheffield, UK  High income country  Multiple setting |
| Study aim | The aim of the project, which was a variation of peer support, was to employ local non-professional mature women to help promote and support breastfeeding within the defined area. |
| Participant information | All breastfeeding women in the area defined for the project |
| Intervention delivery | Multiple intervention delivered in multiple settings |
| Study design (data collection and analysis) | Evaluation study  Face-to-face interviews and audit questionnaire  No information available on how data was analysed |

**Beake 2005a**

| Included |  |
| --- | --- |
| Context (Country and income level, Setting) | London, UK  Urban region; high income country  Multiple settings |
| Study aim | The interviews on which this paper is based formed part of a long-term cohort study to evaluate caseload midwifery practice within a maternity service. Although the focus of the main study was comparing caseload midwifery practice with traditional midwifery practice in the UK, the interviews raised many general issues about post­natal care. Given the lack of published research on women's views of postnatal care, the authors felt these data, warranted further analysis and publication. This paper therefore reports the findings on women's views of postnatal care including their expectations and the type of care they found helpful.  The projects aim, however, was to establish a post of Infant Feeding Support Worker, which would be created and managed within the midwifery service, although funded by Sure Start, on the health care |
| Participant information | Any woman who felt she needed additional support, covering the period from 32 weeks of pregnancy to 4 months postnatally |
| Intervention delivery | Multiple interventions delivered in multiple settings |
| Study design (data collection and analysis) | Mixed longitudinal observational and quasi-experimental  Face-to-face interviews; observations; evaluation study; questionnaires with women, support worker case record forms, midwives record forms; interviews with working group members and focus groups with midwives.  Qualitative data was analysed thematically |

**Beake 2005b**

| Sampled |  |
| --- | --- |
| Context (Country and income level, Setting) | London, UK  Urban region; high income country  Multiple settings |
| Study aim | To contribute to the knowledge on postnatal care in the UK. The interviews on which this paper is based formed part of a long-term cohort study to evaluate caseload midwifery practice within a maternity service. Although the focus of the main study was comparing caseload midwifery practice with traditional midwifery practice in the UK, the interviews raised many general issues about post­natal care. Given the lack of published research on women's views of postnatal care, the authors felt these data, warranted further analysis and publication. This paper therefore reports the findings on women's views of postnatal care including their expectations and the type of care they found helpful.  The projects aim, however, was to establish a post of Infant Feeding Support Worker, which would be created and managed within the midwifery service, although funded by Sure Start, on the health care assistant scale. |
| Participant information | women that were included in a larger trial and randomised to two study arms: women receiving traditional midwifery care vs women receiving caseload care (less continuity and community partners). Women were 8-12 months postnatal |
| Intervention delivery | Multiple intervention delivered in multiple settings |
| Study design (data collection and analysis) | Grounded Theory  Face-to-face interviews |

**Beake 2010**

| Included |  |
| --- | --- |
| Context (Country and income level, Setting) | UK  High income country  In-patient care |
| Study aim | This paper presents data from 20 women who were interviewed on two postnatal wards as part of a quality improvement study, informed by a model of continuous quality improvement. The study aimed to identify where routine systems and processes of care in hospital following birth could be revised to enhance women’s experiences, promote care tailored to need and promote a continuum of care from labour ward to transfer home. |
| Participant information | first-time mothers  women already having children  age 23-39  ethnicity: white European; one woman was Afro-Caribbean and one was Chinese  other: women were recruited at the time they were receiving in-patient postnatal care (n=20); Over half of the women who agreed to take part had had an emergency caesarean section birth (12/20), three women had a planned caesarean birth, two women a spontaneous vaginal birth and three women an assisted vaginal birth. |
| Intervention delivery | Multiple interventions delivered in-patient care |
| Study design (data collection and analysis) | mixed methods with qualitative approach (interviews) and quantitative (pre and post- intervention survey of women at 10 days and 3 months after giving birth)  Face-to-face interviews  Data analysed using content analysis |

**Breedlove 2005**

| Sampled |  |
| --- | --- |
| Context (Country and income level, Setting) | USA  High income country  Multiple settings |
| Study aim | The purpose of this descriptive study was to explore and describe pregnant teens' perceptions of social support provided by ethnically similar, community-based doulas serving in an extended relationship from early pregnancy into early mothering. Four research questions included 1. How do teen women describe networks and individuals that provide assistance and support? 2. What supportive characteristics from the doula are described by teens during pregnancy, labor/ birth, and early mothering? 3. How is support from the doula different from other types of support? 4. How is the support from the doula valued? |
| Participant information | cohort of pregnant (n = 12) and parenting (n = 12) teens  Both primiparous and multiparous  14-18 years  Receiving state Medicaid benefits, reported normal pregnancy or birth, and were involved in the community-based doula project for a minimum of three doula-initiated encounters.  Intendedness of pregnancy, educational level, attendance in school, and employment history |
| Intervention delivery | multiple interventions delivered in multiple settings: Facilitating pregnancy and parenting education classes, providing 24-hour call availability at labour and birth, conducting pre- and post-birth home visitations, and offering additional individual guidance and counselling as needed by the participants. |
| Study design (data collection and analysis) | Descriptive study  Semi-structured interviews  Data analysed using content analysis |

**Bridges 2016**

| Sampled |  |
| --- | --- |
| Context (Country and income level, Setting) | Australia  High-income country  Virtual |
| Study aim | The aim of this study was to advance understanding of the experiences of women using closed Facebook groups attached to the Australia Breastfeeding Association (ABA) and how these women find and share breastfeeding support and information using this forum. This study investigates how breastfeeding women find support online using closed Facebook groups. |
| Participant information | All participants were women with families |
| Intervention delivery | Single intervention delivered virtually |
| Study design (data collection and analysis) | Ethnographic research approach (netnography)  Data collected via: online in-depth interviews (using Facebook 'Chat' function) with administrators of three ABA closed Facebook groups;  online focus groups (using Facebook 'Events' function) of groups of six to eight active participants from each of these three groups; initial observations of these closed ABA facebook groups.  Data analysed thematically. |

**Bula 2015**

| Sampled |  |
| --- | --- |
| Context (Country and income level, Setting) | Malawi, Africa  Rural region; low-income country  At home |
| Study aim | To explore determinants of EBF using MaiMwana infant feeding peer-counselling intervention conducted in Mchinji, Malawi as a case study. Specifically, in this study I explored the effectiveness of the intervention to help HIV positive women to overcome the barriers and examine people’s experiences and perceptions towards the intervention with respect to HIV and poverty. The three main research questions being addressed throughout this doctoral thesis were as follows: What factors promote or hinder the intention and ability of women including those with HIV to practice EBF for the first 6 months of life in rural Malawi? To what extent did the MaiMwana community-based peer counselling assist women in rural Malawi to overcome the barriers and manage to practice EBF for the recommended 6 months period? How did women, peer counsellors and community members perceive community based peer counselling to promote EBF in light of the HIV epidemic and high levels of poverty? |
| Participant information | Women with specific health conditions (HIV)  Language: local or English  Age: older than 18  Residency: coming from the MaiMwana clusters. |
| Intervention delivery | Multiple component intervention delivered at home |
| Study design (data collection and analysis) | Case studies of two interventions. The intervention was a five-year community-based cluster Randomised Controlled Trial (RCT).  Face-to-face interviews; observations of mother’s visits at the clinic (before interview) and of volunteers’ meetings.  Data was analysed using framework analysis. |

**Burns 2017**

| Included |  |
| --- | --- |
| Context (Country and income level, Setting) | Australia  High-income  Multiple settings |
| Study aim | To explore the similarities and differences in breastfeeding communication styles, and language and practices used, in the first month after birth, by privately practicing midwives (PPM), working in a continuity of care model, and, trained breastfeeding peer support counsellors (PSC) providing support at a national breastfeeding organisation’s community based drop-in lounge. |
| Participant information | PPM  PSC  breastfeeding women |
| Intervention delivery | Multiple component intervention delivered in multiple settings (a drop in lounge and women’s homes). |
| Study design (data collection and analysis) | Discourse analysis  Face-to-face interviews; observations and audio recording of interactions  Data was analysed using framework analysis |

**Chaput 2015**

| Included |  |
| --- | --- |
| Context (Country and income level, Setting) | Calgary, Canada  Urban region; high-income country |
| Study aim | To explore the experiences of nursing women with support they received for breastfeeding in order to better inform and optimize existing breastfeeding supports and interventions |
| Participant information | Women aged 18 or older  Intention to breastfeed before delivery  Women delivered healthy babies  Ethnicity: White, First Nations, South Asian, Chinese, Filipina, Latin American  Household income  Marital status  Education  Parity  Language: English, French, Cantonese, Mandarin, Spanish, Tagalog, Portuguese and Armenian |
| Intervention delivery | No information available |
| Study design (data collection and analysis) | A qualitative inquiry embedded in a larger, mixed-methods, prospective cohort study of the relation between breastfeeding difficulties, breastfeeding support and risk of postpartum depression.  Written journal entries reporting on experiences with breastfeeding support.  Data analysed using thematic content analysis with constant comparison techniques. |

**Condon 2012**

| Sampled |  |
| --- | --- |
| Context (Country and income level, Setting) | Bristol, UK  Urban region; high-income country  Hospital setting |
| Study aim | To explore teenagers’ experiences of the breastfeeding promotion and support delivered by health professionals. |
| Participant information | pregnant teenagers aged 18 years or younger x teenage mothers with babies aged 2 years or younger  ethnicity: White British, Black British, British Asian, White European |
| Intervention delivery | Intervention delivered in hospital |
| Study design (data collection and analysis) | Focus-groups and face-to-face interviews  Data was analysed thematically. |

**Condon 2015**

| Included |  |
| --- | --- |
| Context (Country and income level, Setting) | Bristol, UK  Urban region; high income country  Hospital setting |
| Study aim | To explore the views of Gypsy–Traveller women and grandwomen on infant feeding and health service provision. No studies have looked at weaning practices among Gypsies and Travellers, although these are recognised as being important in relation to continuation of breast- feeding, and to later eating habits which have an impact upon risk of obesity and cardiac disease. |
| Participant information | Ethnicity: Women of Roma, English Gypsy or Irish Traveller with a child aged 3 years or under  Number of children  Teenage mothers: Average age at birth of first child below 20 for some  Average age of women at interview  Any educational qualifications  Travelling |
| Intervention delivery | Multiple component intervention |
| Study design (data collection and analysis) | Qualitative approach  Face-to-face interviews  Data analysed using framework analysis |

**Coreil 1995**

| Included |  |
| --- | --- |
| Context (Country and income level, Setting) | 5 States in the USA  High-income country |
| Study aim | We investigated client and provider views of breastfeeding counseling within the context of a larger project to promote breastfeeding among economically disadvantaged women and adolescents. |
| Participant information | First-time mothers  Mothers of multiples  Low-income women  Age: adult/teen  Parity: prima/multi  Geographic residence: Rural/ urban  Feeding method: breast/ bottle  Ethnicity: Anglo-American and Afro-American |
| Intervention delivery | No information available |
| Study design (data collection and analysis) | Focus groups  Data analysed by identifying thematic categories |

**Craig 2010**

| Sampled |  |
| --- | --- |
| Context (Country and income level, Setting) | Australia  High-income country  Private hospital |
| Study aim | To uncover whether inconsistencies relating to the benefits of antenatal breastfeeding education in the overseas literature are also applicable in the Australian context. The question as to whether or not antenatal breastfeeding education is beneficial is investigated through exploration of the experiences of women accessing antenatal breastfeeding education, birthing and then initiating breastfeeding in an Australian maternity unit. The purpose of this study was to uncover the perceived usefulness of a contemporary antenatal education strategy for women' experience of breast feeding initiation. |
| Participant information | First-time mothers  women attending a private hospital |
| Intervention delivery | multiple component intervention delivered in private hospital unit |
| Study design (data collection and analysis) | Qualitative exploratory, descriptive study.  Face-to-face interviews; prior to the intervention one women was called by phone to question her about her needs and expectations of the workshop. This informed the workshop development.  Data was analysed thematically using constant comparison approach |

**Cripe 2010**

| Sampled |  |
| --- | --- |
| Context (Country and income level, Setting) | Arizona, USA  Urban region; high-income country  Suburban hospital |
| Study aim | The aim was to explore women's experiences with social support and infant feeding methods. The study involves three stages and incorporates both quantitative and qualitative approaches. I describe all of them, but take the liberty to afterwards focus on the first stage only. The first stage consists of participant observation and in-depth interviews with support group participants and is designed to help answer the following question: RQ 1: What kinds of support occur in a support group? The next stage consists of a questionnaire for both breastfeeding and formula- feeding women. The questionnaire is designed to answer the following research questions: RQ 2: What kinds of support do women who breastfeed perceive? RQ 3; In what ways do women who breastfeed differ from women who do not breastfeed? RQ 4: How do women who participate in a breastfeeding support group differ from women who breastfeed without a group? The third stage of the study involved returning to the qualitative data to further explore the meaning of the quantitative findings. |
| Participant information | first-time mothers  Pregnant women  mothers of multiples  Age: 21-43  Parity: prima/multi  Baby age: 6 weeks to 13 months  Ethnicity: Caucasian |
| Intervention delivery | Single component intervention delivered in suburban hospital |
| Study design (data collection and analysis) | Narrative studies; exploratory mixed methods design  Face-to-face interviews and observations  No information available on how data was analysed. |

**Cross-Barnet 2012**

| Sampled |  |
| --- | --- |
| Context (Country and income level, Setting) | Maryland, USA  Urban, suburban and semi-rural region; high-income country  Multiple settings |
| Study aim | There is a dearth of research regarding women’ own reports of their experiences with breastfeeding and the medical community, and researchers have recommended examining women’ breastfeeding experiences within their specific social contexts. This paper analyses in-depth, self-reported experiences of WIC participants regarding the prenatal, in-hospital, and paediatric breastfeeding education and support they received beginning in pregnancy and continuing through their child’s infancy. It examines women’ perspectives on the quality and consistency of the information and support they received within each institutional setting as well as across institutions. |
| Participant information | Clients of the peer counselling program  Low socio-economic situation  Women who had an infant and had met at least once with a peer consultant  Mothers of multiples  Women with babies at NICU  Race: black, white  Age: 18-42  Age of infant: 6 days - 12 months  Education: Ninth grade - Masters degree  No of children: 1-6 |
| Intervention delivery | No information on intervention component. Delivered in multiple settings. |
| Study design (data collection and analysis) | Programme evaluation study  Face-to-face interviews  No information available on how data was analysed |

**Da Rocha 2013**

| Sampled |  |
| --- | --- |
| Context (Country and income level, Setting) | Rio de Janeiro, Brazil  Urban region; middle-income country  NICU setting |
| Study aim | To analyse the effect of nursing guidelines on the learning and practice of breastfeeding at the time of the newborn infant’s discharge from the intensive care unit. |
| Participant information | sample characteristics indicated  • first-time mothers  [Info] 13 women were primiparous and 2 were multiparous.  • women having babies with specific (health) conditions please describe in info box  [Info] Women of premature newborn infants from 34 to 36 weeks who were admitted to the ICU and were discharged  • ethnicity_please copy and paste  [Info] Women were living in Rio de Janerio, Brazil, however, it is not stated if they were Brazilian  • other please describe in the info box  [Info] The following were excluded: women whose children had cleft palate, syndromes, and diseases which hindered breast suckling; extremely premature infants; and those cases in which women were living with HIV, because, in these cases, their children couldn’t be breastfed. |
| Intervention delivery | No information regarding intervention component. Delivered in NICU setting. |
| Study design (data collection and analysis) | Descriptive exploratory study  A form for characterisation of subjects with socioeconomic data and an interview script with three open questions |

**Engstrom 2000**

| Sampled |  |
| --- | --- |
| Context (Country and income level, Setting) | Sweden  High-income country |
| Study aim | The aim of this study was to generate a theoretical model of the experiences of women, who had undergone reduction mammoplasty, of counselling received in connection with breast-feeding. |
| Participant information | Women with specific health condition (reduction mammoplasty=breast reduceed)  Women who gave birth to one child  Mothers of multiples  Education  Age  Other: Residence, age of operation, years after operation, marital status |
| Intervention delivery | No information available |
| Study design (data collection and analysis) | Grounded theory  Face-to-face interviews.  Data analysed using constant comparison analysis |

**Entwistle 2010**

| Included |  |
| --- | --- |
| Context (Country and income level, Setting) | UK  High-income country  Maternity unit and community environment |
| Study aim | The aim of this paper is to present the findings of the qualitative interviews and to explore the experiences described by the women from low-income groups within the explanatory framework of the self-efficacy theory. The qualitative study is part of a larger study that examined the impact of the United Nations Children's Fund/UK breast feeding training programme on midwives’ knowledge and attitude and on breastfeeding outcomes for low-income women.  The question = what are the views and experiences of low-income women (defined by Jarman scores) in relation to their breastfeeding support received in the post-natal period? |
| Participant information | First-time mothers  Mothers of multiples  Language: English (at least as second language)  Other: women were purposefully chosen based on a specific SES-index from the larger pool of survey participants; age of baby, breastfed before, breastfeeding at the time of interview, sex of baby, form of delivery, first child of current relationship |
| Intervention delivery | No information available on intervention components. Delivered in maternity unit and community environment. |
| Study design (data collection and analysis) | Face-to-face interviews  Data analysed using thematic analysis |

**Fox 2015**

| Sampled |  |
| --- | --- |
| Context (Country and income level, Setting) | Various locations, UK  Urban and rural region; high-income country  Baby Cafés |
| Study aim | This paper focuses on the qualitative experiences of UK users of Baby Cafe services to examine their experiences of breastfeeding and breastfeeding support. |
| Participant information | First-time mothers  Mothers of multiples  Age (23-44)  Education (no - postgraduate)  Employment (full time - unemployed)  Ethnicity: Asian, Black, White British)  Other: place of birth, age of baby |
| Intervention delivery | Single component intervention delivered in different Baby Cafés |
| Study design (data collection and analysis) | Focus groups, face-to-face interviews and via telephone.  No information available on how data was analysed. |

**Gill 2001**

| Included |  |
| --- | --- |
| Context (Country and income level, Setting) | Florida, USA  High-income country  Maternity Unit |
| Study aim | To ascertain how nurses support breastfeeding women in the hospital and how the women perceived the support they received from the nurses. |
| Participant information | Age: 20 - 38  Most first-time mothers  Mothers of multiples  Education: high school graduation - college  Ethnicity: Hispanic, White, Asian American  Other Annual family income: 15.000 - 50.000 $, marital status, breastfed before, form of birth |
| Intervention delivery | No information on intervention component. Delivered in maternity unit. |
| Study design (data collection and analysis) | Ethnography  Face-to-face interviews and observations  Data was categorised |

**Hailes 2000**

| Included |  |
| --- | --- |
| Context (Country and income level, Setting) | Adelaide, Australia  Urban and rural region; high-income country |
| Study aim | To explore perceptions of available breastfeeding support in the first month postpartum for women in South Australia. |
| Participant information | First-time mothers  Mothers of multiples  Language: English  Others: women who had delivered within the previous six months, had breastfed for some time in the first month following the birth of their baby, attended existing new parent education groups at local child health centres |
| Intervention delivery | No information about intervention components. Delivered in hospital and at home. |
| Study design (data collection and analysis) | Focus-groups  Data was analysed thematically. |

**Hall 2014**

| Included |  |
| --- | --- |
| Context (Country and income level, Setting) | Victoria, Australia  Urban and rural region; high-income country |
| Study aim | To examine factors that inﬂuence the establishment and continuation of breastfeeding among women living in a southern region of Victoria. |
| Participant information | Information not available for focus group participants. |
| Intervention delivery | Multiple component intervention delivered in hospital, home and MCHN centres. |
| Study design (data collection and analysis) | Sequential quantitative and qualitative methods.  Focus groups  Data was analysed thematically. |

**Hoddinott 2006**

| Included |  |
| --- | --- |
| Context (Country and income level, Setting) | Scotland, UK  Rural region; high-income country  Primary care setting or at home |
| Study aim | The aim of the current study was to explore women’s perceptions of one-to-one and group-based breastfeeding peer coaching and to investigate why groups were more popular. |
| Participant information | First-time mothers  Age: 18-44  Pregnant women  Others: woman attending a breastfeeding group, age of baby, Duration of any breastfeeding,  None – 8 mo, Still breastfeeding at time of data collection, Age at leaving full time education (yr), Coaching participation, Participated in one-to-one coaching. Group participation: Attended groups antenatally and/ or postnatally or no groups |
| Intervention delivery | Multiple component intervention delivered in primary care setting or at home. |
| Study design (data collection and analysis) | Multi-methods action research  Focus groups, face-to-face interviews, observations and responses to open-ended questions  Data was analysed using framework and discourse analysis. |

**Hong 2003**

| Sampled |  |
| --- | --- |
| Context (Country and income level, Setting) | USA  High-income country  Maternity unit |
| Study aim | The purpose of this study was to document the perceptions of first-time mothers regarding the breastfeeding support they received from nurses within the first 48 hours after giving birth. |
| Participant information | First-time mothers  Age: 19-33  Ethnicity: Caucasian, Hispanic, Pacific Islander.  Education: completion of high school (12 years) to the first year of graduate school (17 years)  Other: had initiated breastfeeding their healthy, full-term infants. Gave birth vaginally. |
| Intervention delivery | Multiple component intervention delivered in maternity unit |
| Study design (data collection and analysis) | Phenomenology  Face-to-face interviews  No information available on how data was analysed. |

**Hunt 2017**

| Included |  |
| --- | --- |
| Context (Country and income level, Setting) | Cornwall, UK  High-income country  Children’s clinic |
| Study aim | To elicit the reasons for non-access among women, health pro- fessionals and peer supporters within Cornwall, a speciﬁc geographical region in South-West England. the sampling strategy aimed to exhaust theoretical ideas associated with non-access to bf-support. |
| Participant information | First-time mothers  Mothers of multiples  Age: 20-40+  Ethnicity: Caucasian, Hispanic, Pacific Islander.  Education: no formal - degree level  Other: age of baby at time of interview, infant feeding method at time of interview |
| Intervention delivery | Single component intervention delivered in children’s clinic. |
| Study design (data collection and analysis) | Constructionist Grounded Theory  Focus groups, face-to-face interviews and via telephone  Data was analysed using a constant comparison approach |

**Hunter 2015**

| Included |  |
| --- | --- |
| Context (Country and income level, Setting) | Oxfordshire, UK  Urban and rural region; high-income country  Maternity ward |
| Study aim | To explore how the inpatient experiences of a group of young women who gave birth as teenagers influenced their feeding decisions and experiences, and ascertain their ideals for breastfeeding support. |
| Participant information | Teenage mothers  Pregnant  Age: aged 16 or over and had  given birth at age 19 or under  Language: English  Ethnicity: White British, White/Black African Portuguese.  Education: completed education and planned to return to school or college.  Other: had considered breastfeeding or breastfed, place of residence: city, village, rural towns; age of giving birth: 15 years and 11 months, and 19 years and 9 months; age of babies: 2 weeks to 21 months; duration of breastfeeding; breastfeeding vs. no breastfeeding |
| Intervention delivery | No information available on intervention component delivered in maternity ward, unknown for peer support. |
| Study design (data collection and analysis) | Focus-group and face-to-face interviews  Data was analysed using thematic analysis |

**Ingram 2013**

| Included |  |
| --- | --- |
| Context (Country and income level, Setting) | Bristol, UK  Urban region; high-income country  Home and breastfeeding support groups |
| Study aim | This evaluation documented the effects of the peer support service on breastfeeding rates and explored the perceptions of women, midwives and peer supporters. |
| Participant information | Other: Mothers in low prevalence breastfeeding regions of Bristol UK, who had been targeted by a peer support program |
| Intervention delivery | Multiple component intervention delivered at home and breastfeeding support groups |
| Study design (data collection and analysis) | Concurrent triangulation mixed method with survey and qualitative component  Face-to-face and telephone interviews  Data analysed thematically |

**Islam 2016**

| Sampled |  |
| --- | --- |
| Context (Country and income level, Setting) | London, UK  Urban region; high-income country  Home |
| Study aim | Study seeks to increase breast feeding rates among most deprived populations in East-London through peer support networks. However, uptake of this support is low and the study seeks to find out why with a process evaluation. |
| Participant information | All women who had initiated bf and were still bf at the time of their 48-hour postnatal call  Women of the two relevant areas  First-time mothers  Ethnicity: white British  Socio-economic income: low  Age  Other: school leaving age, owning a property, first to bf in their own family  Three groups: those who had declined support, those that had some support but declined a peer supporter and those that had all support forms |
| Intervention delivery | Single component intervention delivered at home |
| Study design (data collection and analysis) | Evaluation study  Face-to-face interviews  Data analysed using thematic content analysis. |

**Johnson 2016**

| Included |  |
| --- | --- |
| Context (Country and income level, Setting) | Detroit, USA  Urban region; high-income country |
| Study aim | To explore African American women's breastfeeding thoughts, attitudes, and experiences with healthcare professionals and subsequent influences on their breastfeeding interest and behaviour. Insight was also sought about the most effective practices to provide breastfeeding support to African American women. |
| Participant information | women or expectant  women who breastfed or planned to do so  women who did not to breastfeed  Ethnicity: African American women  Other: Age in years, marital status, Household  annual income, Education completed |
| Intervention delivery | No information available on intervention component or setting |
| Study design (data collection and analysis) | Focus groups  Data analysed using thematic analysis |

**Leahy-Warren 2017**

| Sampled |  |
| --- | --- |
| Context (Country and income level, Setting) | Ireland  High-income country  Primary Health Care Centre |
| Study aim | To explore breastfeeding women's experience of a public health nurse lead support group |
| Participant information | First-time mothers  Mothers of multiples  Age: 25-35 years  Education: third level education,  Other: marital status: married; form of birth: vaginal delivery and the remaining two had caesarean sections, one elective; breastfeeding before; age of infants: 3 months to 14 months; breastfeeding at time of interview |
| Intervention delivery | Single component intervention delivered in primary health care centre |
| Study design (data collection and analysis) | Qualitative descriptive design  Face-to-face interviews  Data analysed using thematic content analysis |

**Locklin 1994**

| Included |  |
| --- | --- |
| Context (Country and income level, Setting) | Chicago, USA  Urban region; high-income country |
| Study aim | What are the breastfeeding experiences of low-income, minority, urban women who are supported by peer counsellors? Objectives: To attempt to gain insight into personal characteristics that may influence breastfeeding success and to describe the breastfeeding endeavours of a select group of low-income, minority, urban women who received support from trained peer counsellors. The role of the peer counsellor, based on the personal experiences of the participants, was also examined. |
| Participant information | Ethnicity: other than Caucasian--> African American and Latina  Language: English  Education: high school - college degree  Socio-economic background: Low socioeconomic status as defined by enrolment in WIC.  Occupation: unemployed - employment  Other: bf for at least 3 months; having received or in the process of receiving support from a trained peer counsellor; marital status; work status: home vs. part-time vs working; length of bf: 3 months - 2 years, place of residence: inner city; experience of violence |
| Intervention delivery | Multiple component intervention.  No information available on the setting in which it was delivered. |
| Study design (data collection and analysis) | Grounded Theory  Face-to-face interviews  Data analysed using constant comparison analysis |

**MacVicar 2017**

| Included |  |
| --- | --- |
| Context (Country and income level, Setting) | Scotland, UK  High-income country  Tertiary maternity hospital |
| Study aim | The aim of this study was to explore the views of women with opiate dependence on proposed elements for inclusion in a breastfeeding support intervention. |
| Participant information | Women with specific health condition: opiate maintained during pregnancy  Language: English  Age: 16 years of age or over  Ethnicity: White British  Socio-economic background: disadvantaged  Other: women within 6 months post birth, initiated breastfeeding and accessed in-hospital breastfeeding support, roomed-in |
| Intervention delivery | Multiple component intervention delivered in tertiary maternity hospital |
| Study design (data collection and analysis) | Think-aloud technique  Pictorial representations of intervention elements as prompts symbolised practical assistance; one-to-one dedicated sessions’ emotional support; person-centred care and environmental modifications and consolation equipment. Pictorial representations of proposed intervention elements.  Data was analysed using framework analysis |

**McFadden 2013**

| Included |  |
| --- | --- |
| Context (Country and income level, Setting) | West Yorkshire and North East England, UK  High-income country  Multiple setting |
| Study aim | To explore the extent to which cultural context makes a difference to experiences of breast-feeding support for women of Bangladeshi origin and to consider the implications for the provision of culturally appropriate care. |
| Participant information | First-time mothers  Mothers of multiples  Age: maternal age  Language  Education  Socio-economic status  Employment  Other: had breastfed within the previous 5 years; birth place, length of time in the UK, parity and age of youngest child, age of migration, household structure |
| Intervention delivery | Single component intervention delivered in more than one setting |
| Study design (data collection and analysis) | Face-to-face interviews  Data analysed using framework analysis |

**Meier 2007**

| Sampled |  |
| --- | --- |
| Context (Country and income level, Setting) | Michigan, USA  High-income country  Hospital, home and WIC clinic |
| Study aim | The purpose of this project was to complete a qualitative evaluation of the MTMPCP from the perspective of program participants and peer counsellors. We planned to identify components contributing to the efficacy of the program and potential program improvement and/or expansion suggestions. |
| Participant information | Ethnicity: African American/non-Hispanic, white/non-Hispanic, Hispanic.  Age: between 20 and 30 years of age or older. |
| Intervention delivery | Multiple component intervention delivered in hospital, home and WIC clinic |
| Study design (data collection and analysis) | Qualitative evaluation  Focus groups  No information available on how data was analysed |

**Muller 2009**

| Included |  |
| --- | --- |
| Context (Country and income level, Setting) | Sao Paolo, Brazil  Urban region; middle-income country |
| Study aim | To get to know the Social Representations of a group of breastfeeding women about support for breastfeeding and identify actions in the social environment these women perceive as supportive in their breastfeeding processes. [Study findings are broader than formal breastfeeding support – they also include ‘discourses’ on Husbands as companions; the establishment of support networks; support for breastfeeding after a return to work; and general perceptions about bf support – this broader material is not recorded here. |
| Participant information | Age: 19-38  Education: mean of 7,5 years  Employment: paid vs unemployed vs housewife  Family income: 400 - 1,200 reais  Breastfeeding: exclusive breastfeeding (EB),  predominant breastfeeding (PB)  breastfeeding (B) |
| Intervention delivery | Single component intervention delivered in hospital and home |
| Study design (data collection and analysis) | Face-to-face interviews  Collective subject discourse analysis |

**Nankunda 2010**

| Included |  |
| --- | --- |
| Context (Country and income level, Setting) | Uganda, Africa  Urban and rural region; low-income country |
| Study aim | To describe women’s experiences of peer counselling for exclusive breastfeeding in an East African setting. [N.B. This data extraction focuses only on the qualitative findings reported in this paper. The paper also reports quantitative findings from fixed-response interview questions. Both of these sets of findings are part of a broader multi-centre community randomised trial to evaluate the effect of peer counselling for exclusive breastfeeding on infant health. |
| Participant information | First-time mothers  Mothers of multiples  Age: 15-46  Education level: none - secondary and above  Marital status: single and other vs married  Occupation: housewife, farmer, other  Other: about seven months pregnant or within the first week after delivery, no intentions of leaving the study area for at least two years and had to be planning to breastfeed the baby. |
| Intervention delivery | Single component intervention delivered at home |
| Study design (data collection and analysis) | Mixed-methods design  Face-to-face interviews  Data analysed thematically |

**Noble-Carr 2012**

| Sampled |  |
| --- | --- |
| Context (Country and income level, Setting) | New South Wales, Australia  High-income country |
| Study aim | To explore, and gain an understanding of, younger women’ breastfeeding experiences. In particular … how, when and where they prefer to access breastfeeding information and support and what improvements they would like to see in terms of provision of breastfeeding information and support for younger women both now and into the future. [This data extraction focuses only on the qualitative findings about formal BF services reported in this paper. The paper also reports other findings, including two sections headed ‘the stigmatised experience of being a ‘young mum’’, ‘breastfeeding: not a cultural norm’, and ‘Importance of informal networks’, which have not been copied here unless there is reference to a formal service. |
| Participant information | First-time mothers  Mothers of multiples  Age: Mean 20, below 26 as inclusion criteria |
| Intervention delivery | Multiple component intervention.  No information available on setting. |
| Study design (data collection and analysis) | Participatory approach  Focus groups  No information available on how data was analysed. |

**Rossman 2010**

| Sampled |  |
| --- | --- |
| Context (Country and income level, Setting) | Chicago, USA  Urban region; high-income country |
| Study aim | The purpose of this study is to describe women' experiences with certified BPCs related to providing their milk for their VLBW infants in the NICU. Specifically, we sought to understand the maternal perceptions of the relationship that developed between women of VLBW infants and BPCs who had personal knowledge and experiences of providing milk for a VLBW infant and who practiced in an expanded role within a framework of other structured lactation interventions. |
| Participant information | Women of babies with specific health condition  First-time mothers  Mothers of multiples  Age: at least 18 years of age  Ethnicity: Black, White, Latina  Marital status: Married vs not married  Education: High school or less vs Some college  Economic Status: WIC eligible vs Non-WIC eligible  Employment: employed full-time vs Employed part-time  Language: able to speak and understand English  Breastfeeding experience: prior vs no  Prenatal Feeding Plans: Breast-feeding vs Formula-feeding  Feeding plans after Peer Counsellor contact: Breast-feeding only vs Breast-feeding/HM in bottles vs HM/formula in bottles vs Formula-feeding only  Breast-feeding/Pumping: Support at Home  Other: was the mother of a VLBW infant (<1,500 g) hospitalised in the NICU, were at least two weeks postpartum, had met with the breast-feeding peer counsellors at least three times, and had an infant that was expected to survive. |
| Intervention delivery | Multiple component intervention delivered in a Level III NICU Centre. |
| Study design (data collection and analysis) | Qualitative descriptive design  Face-to-face interviews  Data-derived content analysis |

**Sheehan 2009**

| Included |  |
| --- | --- |
| Context (Country and income level, Setting) | NSW Central Coast and Sydney, Australia  Urban region; high-income country  Hospital |
| Study aim | This study examines women's expectations and experiences of infant feeding support provided by health professionals in the first 6 weeks post-birth. The findings are drawn from a grounded theory study exploring women's infant feeding decisions in the first 6 weeks post-birth. |
| Participant information | various different socio-demographic backgrounds, various parity. All women spoke fluent English.  Various parity (primiparas and multiparas)  Age (age and mean: 30)  Partner status (partnered vs separated)  Level of education (University degree, diploma, secondary school year)  Health insurance (private vs public health cover) (Medicare)  Maternity care provider (Private hospital vs Public hospital) |
| Intervention delivery | Single and multiple component intervention delivered in hospital |
| Study design (data collection and analysis) | Grounded theory  Face-to-face interviews  Data analysed using constant comparative method |

**Thomson 2012**

| Sampled |  |
| --- | --- |
| Context (Country and income level, Setting) | England  High-income country |
| Study aim | In this paper, we draw upon the theoretical and practical insights offered by Morse and colleagues to describe how breastfeeding peer support service facilitated and encouraged hope for women’s breastfeeding goals. … This paper reports on data collected with 47 women who had accessed the Star Buddies service…. This [is part of a broader] study [which] was a qualitative exploratory evaluation, utilising focus groups and in-depth interviews. The key aims of the evaluation were to explore health professionals’, peer supporters’ and women’s experiences, facilitators, barriers and challenges faced in the introduction of a breastfeeding peer support service. |
| Participant information | First-time mothers  Mothers of multiples  Age: between 19 and 39 years of age  Status: partners/in relationships vs single vs married  other: age of infant: between 2 weeks and 17 months; feeding: bottle-feeding vs breastfeeding vs mixed feeding; still accessing the community  Star Buddies service at the time of the interview vs stopped the service early because of breastfeeding cessation vs had received at least 8 weeks of community breastfeeding support. |
| Intervention delivery | Multiple component intervention delivered in clinic, maternity ward and home. |
| Study design (data collection and analysis) | Qualitative evaluation  Face-to-face interviews and telephone interviews  Data analysed using framework analysis |

**Thomson 2013**

| Included |  |
| --- | --- |
| Context (Country and income level, Setting) | England  High-income country |
| Study aim | To report on the descriptive and qualitative insights provided by 908 callers as part of an evaluation of UK-based breastfeeding helpline(s). Concretely, callers’ experiences of the help and support received via the breastfeeding helpline(s) was explored. |
| Participant information | First-time mothers  Mothers of multiples  Age: between under 20 and older than 40  Status: partners/in relationships vs single vs married  other: age of infant: pregnant, under 1 month, between 1 and 12 months, over 12 months; feeding: bottle-feeding vs breastfeeding vs mixed feeding |
| Intervention delivery | Breastfeeding peer support in the form of a breastfeeding helpline |
| Study design (data collection and analysis) | Mixed-method with structured telephone interview and open-ended questions was undertaken with 908 callers over May to August, 2011 |

**Thorstensson 2016**

| Included |  |
| --- | --- |
| Context (Country and income level, Setting) | Sweden  High-income country  Maternity unit |
| Study aim | To illuminate first-time mothers’ experience of professional support at the maternity ward as a step in the validation of the Mother-Perceived-Professional-Support (MoPPS) scale. [This data extraction focuses only on the qualitative findings about BF. The paper also reports: a) findings related to wider aspects of professional care and support in the maternity ward for first-time mothers; b) answers given within a survey tool aimed at measuring women’s ratings of this professional support]. |
| Participant information | First-time mothers  Age: 23 to 42  Other: birth experiences normal birth vs instrumental vaginal birth vs caesarean birth |
| Intervention delivery | No information available on intervention component.  Delivered in maternity unit |
| Study design (data collection and analysis) | Inductive and deductive approach with think aloud technique.  Face-to-face interviews  Data was analysed using content analysis |

**Wade 2009**

| Included |  |
| --- | --- |
| Context (Country and income level, Setting) | Northumberland, UK  High-income country |
| Study aim | Anecdotal discussion among breastfeeding peer supporters and the infant-feeding co-ordinator suggested that breastfeeding peer support provided by breastfeeding peer supporters may offer benefits to breastfeeding women and their families other than increasing breastfeeding initiation and sustainability. The aim of this research was to determine whether there was evidence to support this. |
| Participant information | Women who had been in contact with breastfeeding peer supporters in Northumberland UK  Children's age: 2 months - 3 years |
| Intervention delivery | Multiple component intervention delivered at home, support groups. |
| Study design (data collection and analysis) | Focus groups  Data was analysed thematically |

**Weimers 2006**

| Sampled |  |
| --- | --- |
| Context (Country and income level, Setting) | Stockholm, Sweden  Urban region; high-income country  Maternity unit |
| Study aim | How do women experience hands-on support by nursing staff in breastfeeding situations while their baby is in a NICU? [This was a sub-question of the broader qualitative study on which this paper reports. The broader study’s question was “How do women experience support by nursing staff in breastfeeding situations while their baby is in the NICU?] The aim was to reveal Swedish women' experiences of hands-on nursing approach to support breastfeeding initiation in a NICU |
| Participant information | First-time mothers  Mothers of multiples  Women of babies having health conditions (sick)  Women of premature born babies  Women of twins  Language: Swedish, at least understanding it  Age 26-35  Other: women experiencing hands-on support,  have given birth to either a premature infant or a sick baby requiring to be hospitalised in the NICU, and to be breastfeeding.  Also, according to the inclusion criteria, 50% of the babies should be full term sick infants and 50% premature |
| Intervention delivery | No information available on intervention component. Delivered in maternity unit. |
| Study design (data collection and analysis) | Face-to-face interviews  Data was analysed using Radnitzky's principles for hermeneutic interpretation |

**Whelan 2014**

| Included |  |
| --- | --- |
| Context (Country and income level, Setting) | North Dublin, Ireland  Urban and sub-urban region; high-income country  Antenatal, hospital and community |
| Study aim | To examine women’s experience of professional support for breastfeeding and health-care professionals’ experience of providing support |
| Participant information | First-time mothers  Mothers of multiples  Education: secondary vs. tertiary level  Age: 25 and older  Other: feeding exclusive bf vs. complementary feeding (breast milk and any other food or liquid, including formula, given at 6 months of age or beyond). |
| Intervention delivery | No information available on intervention component. Delivered in antenatal, hospital and community setting. |
| Study design (data collection and analysis) | Face-to-face interviews  Data analysed using content analysis and grounded theory approach |

### References to studies meeting eligibility

Ahluwalia IB, Tessaro I, Grummer-Strawn LM, MacGowan C, Benton-Davis S. Georgia's breastfeeding promotion program for low-income women. Pediatrics. 2000 Jun;105(6):E85. doi: 10.1542/peds.105.6.e85. PMID: 10835098.

Andaya E, Bonuck K, Barnett J, Lischewski-Goel J. Perceptions of primary care-based breastfeeding promotion interventions: qualitative analysis of randomized controlled trial participant interviews. Breastfeed Med. 2012 Dec;7(6):417-22. doi: 10.1089/bfm.2011.0151. Epub 2012 May 23. PMID: 22621223; PMCID: PMC3523239.

Andreson J, Dana N, Hepfer B, King'ori E, Oketch J, Wojnar D, Cowgill K, Israel-Ballard K. Infant feeding buddies: a strategy to support safe infant feeding for HIV-positive mothers. J Hum Lact. 2013 Feb;29(1):90-3. doi: 10.1177/0890334412469056. PMID: 23277462.

Bäckström CA, Wahn EI, Ekström AC. Two sides of breastfeeding support: experiences of women and midwives. Int Breastfeed J. 2010 Nov 29;5:20. doi: 10.1186/1746-4358-5-20. PMID: 21114812; PMCID: PMC3001698.

Bailey S. Postnatal care: exploring the views of first-time mothers. Community Pract. 2010 Dec;83(12):26-9. PMID: 21214136.

Barona-Vilar C, Escribá-Agüir V, Ferrero-Gandía R. A qualitative approach to social support and breast-feeding decisions. Midwifery. 2009 Apr;25(2):187-94. doi: 10.1016/j.midw.2007.01.013. Epub 2007 May 9. PMID: 17493716.

Battersby S. The Worldly Wise project. A different approach to breastfeeding support. Pract Midwife. 2001 Jun;4(6):30-1. PMID: 12026844.

Beake, S, McCourt C, Bick D. Women's views of hospital and community-based postnatal care: the good, the bad and the indifferent. Evidence Based Midwifery, 2005 3 (2). pp. 80-86. ISSN 1479-4489

Beake S, McCourt C, Rowan C, Taylor J. Evaluation of the use of health care assistants to support disadvantaged women breastfeeding in the community. Matern Child Nutr. 2005 Jan;1(1):32-43. doi: 10.1111/j.1740-8709.2004.00007.x. PMID: 16881877; PMCID: PMC6874387.

Beake S, Rose V, Bick D, Weavers A, Wray J. A qualitative study of the experiences and expectations of women receiving in-patient postnatal care in one English maternity unit. BMC Pregnancy Childbirth. 2010 Oct 27;10:70. doi: 10.1186/1471-2393-10-70. PMID: 20979605; PMCID: PMC2978124.

Breedlove G. Perceptions of social support from pregnant and parenting teens using community-based doulas. J Perinat Educ. 2005 Summer;14(3):15-22. doi: 10.1624/105812405X44691. PMID: 17273438; PMCID: PMC1595255.

Bridges N. The faces of breastfeeding support: Experiences of mothers seeking breastfeeding support online. Breastfeed Rev. 2016 Mar;24(1):11-20. PMID: 27188074.

Bula Agatha Kapatuka (2015). Influences of HIV on exclusive breastfeeding: an exploration of community-based peer support in rural Malawi. (Unpublished Doctoral thesis, City University London)

Burns E, Schmied V. "The right help at the right time": Positive constructions of peer and professional support for breastfeeding. Women Birth. 2017 Oct;30(5):389-397. doi: 10.1016/j.wombi.2017.03.002. Epub 2017 Mar 27. PMID: 28359753.

Chaput KH, Adair CE, Nettel-Aguirre A, Musto R, Tough SC. The experience of nursing women with breastfeeding support: a qualitative inquiry. CMAJ Open. 2015 Jul 17;3(3):E305-9. doi: 10.9778/cmajo.20140113. PMID: 26442229; PMCID: PMC4593410.

Condon LJ, Withall J, Warren S, Tapp A. But is it a normal thing?' Teenage mothers' experiences of breastfeeding promotion and support. Health Education Journal 2012 72(2):156-162

Condon LJ, Salmon D. 'You likes your way, we got our own way': Gypsies and Travellers' views on infant feeding and health professional support. Health Expect. 2015 Oct;18(5):784-95. doi: 10.1111/hex.12214. Epub 2014 Jun 3. PMID: 24890123; PMCID: PMC5060882.

Coreil J, Bryant CA, Westover BJ, Bailey D. Health professionals and breastfeeding counseling: client and provider views. J Hum Lact. 1995 Dec;11(4):265-71. doi: 10.1177/089033449501100411. PMID: 8634102.

Craig HJ, Dietsch E. 'Too scary to think about': first time mothers' perceptions of the usefulness of antenatal breastfeeding education. Women Birth. 2010 Dec;23(4):160-5. doi: 10.1016/j.wombi.2010.04.004. Epub 2010 May 20. PMID: 20493795.

Cripe Emily T. (2010) Breastfeeding Support Explored through Mixed Methods: How Support Groups Can Function as an Effective Health Intervention. (Unpublished Doctoral thesis, Arizona State University)

Cross-Barnet C, Augustyn M, Gross S, Resnik A, Paige D. Long-term breastfeeding support: failing mothers in need. Matern Child Health J. 2012 Dec;16(9):1926-32. doi: 10.1007/s10995-011-0939-x. PMID: 22246714.

Da Rocha CR, da Silva LR , Soeiro G , de Oliveira Vasconcellos MR , Abrão DF, da Silva6 LR. Learning and breastfeeding practice in the neonatal intensive care unit: experience of women. (2013) Journal of Nursing UFPE online., Recife, 7(3):641-8

Engström BL, Fridlund B. Women's views of counselling received in connection with breast-feeding after reduction mammoplasty. J Adv Nurs. 2000 Nov;32(5):1143-51. doi: 10.1046/j.1365-2648.2000.01585.x. PMID: 11114999.

Entwistle F, Kendall S, Mead M. Breastfeeding support - the importance of self-efficacy for low-income women. Matern Child Nutr. 2010 Jul 1;6(3):228-42. doi: 10.1111/j.1740-8709.2009.00202.x. PMID: 20929495; PMCID: PMC6860837.

Fox R, McMullen S, Newburn M. UK women's experiences of breastfeeding and additional breastfeeding support: a qualitative study of Baby Café services. BMC Pregnancy Childbirth. 2015 Jul 7;15:147. doi: 10.1186/s12884-015-0581-5. PMID: 26148545; PMCID: PMC4494694.

Gill SL. The little things: perceptions of breastfeeding support. J Obstet Gynecol Neonatal Nurs. 2001 Jul-Aug;30(4):401-9. doi: 10.1111/j.1552-6909.2001.tb01559.x. PMID: 11461024.

Hailes JF, Wellard SJ. Support for breastfeeding in the first postpartum month: perceptions of breastfeeding women. Breastfeed Rev. 2000 Nov;8(3):5-9. PMID: 11210281.

Hall H, McLelland G, Gilmour C, Cant R. 'It's those first few weeks': Women's views about breastfeeding support in an Australian outer metropolitan region. Women Birth. 2014 Dec;27(4):259-65. doi: 10.1016/j.wombi.2014.06.007. Epub 2014 Jul 14. PMID: 25034510.

Hoddinott P, Chalmers M, Pill R. One-to-one or group-based peer support for breastfeeding? Women's perceptions of a breastfeeding peer coaching intervention. Birth. 2006 Jun;33(2):139-46. doi: 10.1111/j.0730-7659.2006.00092.x. PMID: 16732780.

Hong TM, Callister LC, Schwartz R. First time mothers' views of breastfeeding support from nurses. MCN Am J Matern Child Nurs. 2003 Jan-Feb;28(1):10-5. doi: 10.1097/00005721-200301000-00004. PMID: 12514351.

Hunt L, Thomson G. Pressure and judgement within a dichotomous landscape of infant feeding: a grounded theory study to explore why breastfeeding women do not access peer support provision. Matern Child Nutr. 2017 Apr;13(2):e12279. doi: 10.1111/mcn.12279. Epub 2016 Apr 1. PMID: 27037727; PMCID: PMC6865888.

Hunter L, Magill-Cuerden J, McCourt C. Disempowered, passive and isolated: how teenage mothers' postnatal inpatient experiences in the UK impact on the initiation and continuation of breastfeeding. Matern Child Nutr. 2015 Jan;11(1):47-58. doi: 10.1111/mcn.12150. Epub 2014 Sep 25. PMID: 25257851; PMCID: PMC6860278.

Ingram J. A mixed methods evaluation of peer support in Bristol, UK: mothers', midwives' and peer supporters' views and the effects on breastfeeding. BMC Pregnancy Childbirth. 2013 Oct 20;13:192. doi: 10.1186/1471-2393-13-192. PMID: 24139326; PMCID: PMC3854768.

Islam MP. Why are 'hard-to-reach' women not engaging in a breastfeeding peer support programme? Community Pract. 2016 Feb;89(2):36-41. PMID: 27164801.

Johnson AM, Kirk R, Rooks AJ, Muzik M. Enhancing Breastfeeding Through Healthcare Support: Results from a Focus Group Study of African American Mothers. Matern Child Health J. 2016 Nov;20(Suppl 1):92-102. doi: 10.1007/s10995-016-2085-y. PMID: 27449776; PMCID: PMC5290044.

Leahy-Warren P, Creedon M, O'Mahony A, Mulcahy H. Normalising breastfeeding within a formula feeding culture: An Irish qualitative study. Women Birth. 2017 Apr;30(2):e103-e110. doi: 10.1016/j.wombi.2016.10.002. Epub 2016 Nov 5. PMID: 27825777.

Locklin Maryanne Phyllis (1994) Gaining a voice: A study of the breastfeeding experience of a select group of educated, low-income, minority women supported by peer counsellors. (Unpublished Docoral thesis, Rush University)

MacVicar S PhD, Humphrey T PhD, Forbes-McKay KE PhD. Breastfeeding support and opiate dependence: A think aloud study. Midwifery. 2017 Jul;50:239-245. doi: 10.1016/j.midw.2017.04.013. Epub 2017 Apr 27. PMID: 28494389.

McFadden A, Renfrew MJ, Atkin K. Does cultural context make a difference to women's experiences of maternity care? A qualitative study comparing the perspectives of breast-feeding women of Bangladeshi origin and health practitioners. Health Expect. 2013 Dec;16(4):e124-35. doi: 10.1111/j.1369-7625.2012.00770.x. Epub 2012 Mar 20. PMID: 22429489; PMCID: PMC5060684.

Meier ER, Olson BH, Benton P, Eghtedary K, Song WO. A qualitative evaluation of a breastfeeding peer counselor program. J Hum Lact. 2007 Aug;23(3):262-8. doi: 10.1177/0890334407303892. PMID: 17666536.

Müller FS, Silva IA. Social representations about support for breastfeeding in a group of breastfeeding women. Rev Lat Am Enfermagem. 2009 Sep-Oct;17(5):651-7. doi: 10.1590/s0104-11692009000500009. PMID: 19967213.

Nankunda J, Tumwine JK, Nankabirwa V, Tylleskär T; PROMISE-EBF Study Group. "She would sit with me": mothers' experiences of individual peer support for exclusive breastfeeding in Uganda. Int Breastfeed J. 2010 Oct 26;5:16. doi: 10.1186/1746-4358-5-16. PMID: 20977715; PMCID: PMC2987850.

Noble-Carr D, Bell C. Exposed: younger mothers and breastfeeding. Breastfeed Rev. 2012 Nov;20(3):27-38. PMID: 23330448.

Rossman B. (2010) Breastfeeding Peer Counselors in the Neonatal Intensive Care Unit: Maternal Perspectives (Unpublished doctoral thesis, University of Illinois at Chicago)

Sheehan A, Schmied V, Barclay L. Women's experiences of infant feeding support in the first 6 weeks post-birth. Matern Child Nutr. 2009 Apr;5(2):138-50. doi: 10.1111/j.1740-8709.2008.00163.x. PMID: 19292748; PMCID: PMC6860642.

Thomson G, Crossland N, Dykes F. Giving me hope: women's reflections on a breastfeeding peer support service. Matern Child Nutr. 2012 Jul;8(3):340-53. doi: 10.1111/j.1740-8709.2011.00358.x. Epub 2011 Nov 28. PMID: 22118045; PMCID: PMC6860716.

Thomson G, Crossland N. Callers’ attitudes and experiences of UK breastfeeding helpline support. International Breastfeed Journal 2013;8(3):1-15. [DOI: 10.1186/1746-4358-8-3]

Thorstensson S, Andersson A, Israelsson S, Ekström A, Hertfelt Wahn E. To build a bridge between two worlds: Mothers' experiences of professional support at the maternity ward. Health Care Women Int. 2016 Oct;37(10):1067-81. doi: 10.1080/07399332.2015.1094072. Epub 2015 Sep 23. PMID: 26397360.

Wade D, Haining S, Day A. Breastfeeding peer support: are there additional benefits? Community Pract. 2009 Dec;82(12):30-3. PMID: 20067068.

Weimers L, Svensson K, Dumas L, Navér L, Wahlberg V. Hands-on approach during breastfeeding support in a neonatal intensive care unit: a qualitative study of Swedish mothers' experiences. Int Breastfeed J. 2006 Oct 26;1:20. doi: 10.1186/1746-4358-1-20. PMID: 17064423; PMCID: PMC1634844.

Whelan B, Kearney JM. Breast-feeding support in Ireland: a qualitative study of health-care professionals' and women's views. Public Health Nutr. 2015 Aug;18(12):2274-82. doi: 10.1017/S1368980014002626. Epub 2014 Dec 1. PMID: 25435017.
