# Supplementary material for: Factors that influence women's engagement with breastfeeding support: A qualitative evidence synthesis
Source: Matern Child Nutr. 2022 Aug 25;18(4):e13405. doi: 10.1111/mcn.13405 (PMC9480951; doi:10.1111/mcn.13405)
Supplement: Supplementary file 2 — Supplementary information. [file MCN-18-e13405-s003.docx]

### MEDLINE Search Strategy

1. breast feeding/

2. (breast feed* or breastfeed* or lactation).ti,ab,kf.

3. or/1-2

4. (support* or communicat* or messag* or internet or phone? or written or print* or educat* or teach* or train* or coach* or promot* or advis* or advice* or counsel* or advocacy or encourag* or peer?).mp.

5. exp maternal health services/

6. maternal health/

7. maternal welfare/

8. exp professional patient relations/

9. professional family relations/

10. or/4-9

11. 3 and 10

12. qualitative research/

13. qualitative.ti,ab,kf.

14. interview*.mp.

15. focus group*.mp.

16. case stud*.ti,ab,kf.

17. mixed methods.ti,ab,kf.

18. hermeneutic*.mp.

19. phenomenolog*.ti,ab,kf.

20. grounded theory.mp.

21. ethnograph*.ti,ab,kf.

22. or/12-21

23. 11 and 22
