# Supplementary material for: Factors that influence women's engagement with breastfeeding support: A qualitative evidence synthesis
Source: Matern Child Nutr. 2022 Aug 25;18(4):e13405. doi: 10.1111/mcn.13405 (PMC9480951; doi:10.1111/mcn.13405)
Supplement: Supplementary file 3 — Supplementary information. [file MCN-18-e13405-s001.docx]

### Characteristics and references of excluded studies

Characteristics of Excluded Studies (ordered by study ID)

| **Study** | **Reason for exclusion** |
| --- | --- |
| Adewale (2006) | Did not investigate perspectives and/or experiences of support as deﬁned in the review. |
| Agunbiade (2012) | Did not investigate perspectives and/or experiences of support as deﬁned in the review. |
| Ahmed (2006) | Did not investigate perspectives and/or experiences of support as defined in the review. |
| Albers (1981) | Did not investigate perspectives and/or experiences of support as defined in the review. |
| Albuquerque (2013) | Did not investigate support programmes at all. |
| Alexander (2003) | Did not use qualitative methods for data collection and analysis. |
| Alianmoghaddam (2017) | Did not investigate perspectives and/or experiences of support as defined in the review. |
| Almiron (1996) | Did not investigate perspectives and/or experiences of support as defined in the review. |
| Amir (2006) | Did not address support as defined in the review. |
| Anonymous (1985) | Not enough information to be retrieve data. |
| Araujo (2015) | Not enough information to be retrieve data. |
| Arora (1985) | Did not investigate support programmes at all. |
| Arts (2011) | Did not investigate support programmes at all. |
| Asiodu (2014) | Did not investigate support programmes at all. |
| Asiodu (2017) | Did not investigate perspectives and/or experiences of support as defined in the review. |
| Baeza (2016) | Did not investigate support programmes at all. |
| Bailey (2001) | Did not investigate support programmes at all. |
| Bailey (2004) | Did not investigate support programmes at all. |
| Bailey (2007) | Not enough information to retrieve data. |
| Barbieri (2013) | Not able to separate out data on perception on support related to breastfeeding. |
| Barimani (2017) | Did not investigate perspectives and/or experiences of support as defined in the review. |
| Barnes (2013) | Did not investigate support programmes at all. |
| Bar-Yam (1997) | Did not investigate perspectives and/or experience of support as defined in the review. |
| Bergman (1993) | Did not use qualitative methods for data collection and analysis. |
| Bernal (2017) | Not enough information to retrieve data. |
| Bledsoe-Mansori (2013) | Not enough information to retrieve data. |
| Bowman (2013) | Did not investigate support programmes at all. |
| Bradford (2017) | Did not investigate perspectives and/or experiences of support of women but of professionals. |
| Brito (2012) | Not enough information to retrieve data. |
| Brown (2001) | Did not investigate perspectives and/or experiences of support of women but of professionals. |
| Brown (2013) | Did not investigate perspectives and/or experiences of support as defined in the review. |
| Brown (2014) | Did not address support as defined in the review. |
| Bula (2013) | Did not investigate perspectives and/or experiences of support of women but of professionals. |
| Chang (2014) | Did not investigate perspectives and/or experiences of support as defined in the review. |
| Chaput (2016) | Not able to separate out qualitative findings from data. |
| Chin (2013) | Did not investigate support programmes at all. |
| Condon (2011) | Did not address support as defined in the review. |
| de Oliveira (2010) | Not translated due to insufficient translation capacities. |
| Demirtas (2012) | Did not investigate support programmes at all. |
| Demirtas (2012) | This is a SR of which one primary study was included (Sheehan 2009). |
| Demirtas (2015) | Did not use qualitative methods for data collection and analysis. |
| Dykes (2003) | Did not investigate support programmes at all. |
| Fadnes (2010) | Did not address support as defined in the review. |
| Finigan (2003) | Not enough information to retrieve data. |
| Foley (2013) | Did not investigate support programmes at all. |
| Fraser (1997) | Not enough information to retrieve data. |
| Geller (2013) | Did not use qualitative methods for data collection and analysis. |
| Graffy (2005) | Did not use qualitative methods for data collection and analysis. |
| Grassley (2008) | Did not address support as defined in the review. |
| Hall (2007) | This is a SR of which the included qualitative studies did not investigate perception of support. |
| Hauck (2011) | Did not use qualitative methods for data collection and analysis. |
| Hauck (2016) | Did not investigate perspectives and/or experiences of support as defined in the review. |
| Heidari (2017) | Did not investigate support programmes at all. |
| Hunter-Adams (2016) | Did not investigate perspectives and/or experiences of support as defined in the review. |
| Isabella (1994) | Did not investigate perspectives and/or experiences of support as defined in the review. |
| Kantrowitz-Gordon (2017) | Did not investigate support programmes at all. |
| Koerber (2012) | Did not investigate support programmes at all. |
| Laterra (2014) | Did not investigate support programmes at all. |
| Lin (2004) | Did not investigate support programmes at all. |
| MacVicar (2014) | This is a SR of which some of the included qualitative studies were not relevant |
| McInnes (2008) | This is a SR of which the included qualitative studies were not relevant. |
| Nelson (2007) | Did not investigate perspectives and/or experiences of support of women but of professionals. |
| Peritore (2016) | Did not address support as defined in the review. |
| Price (2014) | Did not use qualitative methods for data collection and analysis. |
| Rwami (2009) | Not enough information to retrieve data. |
| Segeel (2006) | Did not investigate support programmes at all. |
| Sercekus (2010) | Not enough information to retrieve data. |
| Sim (2015) | Did not address support as defined in the review. |
| No author information (2010) | Not enough information to retrieve data. |

### References to studies excluded from this review

Adewale, OR. The Lived Experience of First-Time Breastfeeding Mothers. International Journal of Childbirth Education. Sep2006, Vol. 21 Issue 3, p21-25. 5p.

Agunbiade OM, Ogunleye OV. Constraints to exclusive breastfeeding practice among breastfeeding mothers in Southwest Nigeria: implications for scaling up. Int Breastfeed J. 2012 Apr 23;7:5. doi: 10.1186/1746-4358-7-5. PMID: 22524566; PMCID: PMC3359265.

Ahmed, S., Macfarlane, A. J., Naylor, J. and Hastings, J. (2006). Evaluating bilingual peer support for breastfeeding in a local Sure Start. British Journal of Midwifery, 14(8), pp. 467-470. doi: 10.12968/bjom.2006.14.8.21640

Albers RM. Emotional support for the breast-feeding mother. Issues Compr Pediatric Nursing. 1981 Mar-Apr;5(2):109-24. doi: 10.3109/01460868109105389. PMID: 6915033.

Albuquerque MF, Casimiro CF, de Oliveira Bastos P, Albuquerque OSF, Cavalcante Martins M, Soares Gondim AP. Mothers' knowledge concerning breastfeeding and complementation food: an exploratory study. Online Brazilian Journal of Nursing. (2013) 12 (1) https://doi.org/10.5935/1676-4285.20133890

Alexander J, Anderson T, Grant M, Sanghera J, Jackson D. An evaluation of a support group for breast-feeding women in Salisbury, UK. Midwifery. 2003 Sep;19(3):215-20. doi: 10.1016/s0266-6138(03)00033-0. PMID: 12946337.

Alianmoghaddam N, Phibbs S, Benn C. New Zealand women talk about breastfeeding support from male family members. Breastfeed Rev. 2017 Mar;25(1):35-44. PMID: 29211384.

Almirón P, Fatjó A, Fernández M, Fernández de Sanmamed MJ, Gómez MA, Martí M, Relat V. Mujeres y lactancia: entendiendo sus vivencias y analizando la actuación del sistema sanitario [Women and breast feeding: understanding their experiences and analyzing the performance of the health system]. Aten Primaria. 1996 May 15;17(8):501-6. Spanish. PMID: 8679887.

Amir LH, Lumley J. Women's experience of lactational mastitis--I have never felt worse. Aust Fam Physician. 2006 Sep;35(9):745-7. PMID: 16969450.

Anonymous. Rooming-in: keeping mother and infant together. Network Research Triangle Park N C. 1985 Autumn;7(1):1,8. PMID: 12314028.

Araujo R. Improving breastfeeding services for neonates with Congenital Heart Defect using the Experience-Based Co-Design approach. Royal Brompton Hospital, London, United Kingdom. Conference abstract at the annual meeting of the Association for European Paediatric and Congenital Cardiology 2015

Arora AK, Singh RN, Gupta BD, Gupta M, Dabi DR. Social customs and beliefs regarding breast feeding. Indian Pediatr. 1985 Dec;22(12):907-9. PMID: 3837777.

Arts M, Geelhoed D, De Schacht C, Prosser W, Alons C, Pedro A. Knowledge, beliefs, and practices regarding exclusive breastfeeding of infants younger than 6 months in Mozambique: a qualitative study. J Hum Lact. 2011 Feb;27(1):25-32; quiz 63-5. doi: 10.1177/0890334410390039. Epub 2010 Dec 22. PMID: 21177988.

Asiodu, I. V. (2014). Identifying Barriers and Facilitators to Breastfeeding Initiation in the African American Community. UCSF. ProQuest ID: Asiodu_ucsf_0034D_10976.REDACTED. Merritt ID: ark:/13030/m5v99p9z. Retrieved from https://escholarship.org/uc/item/7gr283xd

Asiodu IV, Waters CM, Dailey DE, Lyndon A. Infant Feeding Decision-Making and the Influences of Social Support Persons Among First-Time African American Mothers. Matern Child Health J. 2017 Apr;21(4):863-872. doi: 10.1007/s10995-016-2167-x. PMID: 27565664; PMCID: PMC5329142.

Baeza WB; Henriquez KF, Prieto GR. Parenteral postnatal resting:experience in breast feeding of workers mothers using public health system in Region of Araucania of Chile. Review of child nutrition [online]. 2016, vol.43, n.2, pp.131-137. ISSN 0717-7518. http://dx.doi.org/10.4067/S0717-75182016000200004.

Bailey C, Pain R. Geographies of infant feeding and access to primary health-care. Health Soc Care Community. 2001 Sep;9(5):309-17. doi: 10.1046/j.1365-2524.2001.00308.x. PMID: 11560746.

Bailey C, Pain RH, Aarvold JE. A 'give it a go' breast-feeding culture and early cessation among low-income mothers. Midwifery. 2004 Sep;20(3):240-50. doi: 10.1016/j.midw.2003.12.003. PMID: 15337280.

Bailey J, Modern parents' perspectives on breastfeeding: A small study. British Journal of Midwifery. 2007 15(3):148-152. doi: 10.12968/bjom.2007.15.3.23034.

Bar-Yam, N. Nursing Mothers at Work: Corporate and Maternal Strategies to Support Lactation in the Workplace. Journal of the Motherhood Initiative for Research and Community Involvement (2004)

Barbieri MC , Soares NT ,Ferrari RAP, de Oliveira Demitto M, Tacla MM. The experience of motherhood: perception of women participants from prenatal groups. Journal of Nursing UFPE on line, 2013 Recife 7(9):5533-40

Barimani M, Forslund Frykedal K, Rosander M, Berlin A. Childbirth and parenting preparation in antenatal classes. Midwifery. 2018 Feb;57:1-7. doi: 10.1016/j.midw.2017.10.021. Epub 2017 Oct 31. PMID: 29128739.

Barnes M, Roiko A, Reed R, Williams C, Willcocks K. Experiences of birth and breastfeeding following assisted conception. Breastfeed Rev. 2013 Mar;21(1):9-15. Erratum in: Breastfeed Rev. 2013 Jul;21(2):41. Roiko, Anne [added]; Reed, Rachel [added]; Williams, Cath [added]; Willcocks, Kerry [added]. PMID: 23600323.

Bergman V, Larsson S, Lomberg H, Möller A, Mårild S. A survey of Swedish mothers' view on breastfeeding and experiences of social and professional support. Scand J Caring Sci. 1993;7(1):47-52. doi: 10.1111/j.1471-6712.1993.tb00161.x. PMID: 8502855.

Bernal I J; Garcia-Meza R ; Duque T ; Bonvecchio A ; Cosio I ; Gonzalez W ; Tumilowicz A. Exploring the influence and transfer of infant and young child feeding practices to mothers: Formative research for the spoon project in Colombia, Guatemala and Mexico. IUNS. 21st International Congress of Nutrition. Buenos Aires, Argentina, October 15-20, 2017: Abstracts. Annals of Nutrition and Metabolism 2017;71(suppl 2):1-1433. doi: 10.1159/000480486

Bledsoe-Mansori S E; Andringa K ; Killian C ; Bellows A ; Doernberg A ; Squires M ; Wessel J ; Steube A ; Meltzer-Brody S. Perceptions of breastfeeding success in depressed and non-depressed mothers: Implications for evidence-based practices. The Marcé International Society International Biennial General Scientific Meeting

Bowman, Roxanne K., The factors that influence duration of exclusive breastfeeding: a mixed methods design. 2013 Theses and Dissertations--Nursing. 9. https://uknowledge.uky.edu/nursing_etds/9

Bradford VA, Walkinshaw LP, Steinman L, Otten JJ, Fisher K, Ellings A, O'Leary J, Johnson DB. Creating Environments to Support Breastfeeding: The Challenges and Facilitators of Policy Development in Hospitals, Clinics, Early Care and Education, and Worksites. Matern Child Health J. 2017 Dec;21(12):2188-2198. doi: 10.1007/s10995-017-2338-4. PMID: 28707098.

Brito AAC, Cavalcante RD, Farias TRO. Perception of breastfeeding mothers about the support of primary care nurses on exclusive breastfeeding. Journal of Nursing UFPE on line, 2012 Recife 6(2):483-7

Brown CA, Poag S, Kasprzycki C. Exploring large employers' and small employers' knowledge, attitudes, and practices on breastfeeding support in the workplace. J Hum Lact. 2001 Feb;17(1):39-46. doi: 10.1177/089033440101700108. PMID: 11847850.

Brown LF, Pickler R. A guided feeding intervention for mothers of preterm infants: two case studies. J Spec Pediatr Nurs. 2013 Apr;18(2):98-108. doi: 10.1111/jspn.12020. Epub 2013 Mar 24. PMID: 23560581.

Brown S, Brage Hudson D, Campbell-Grossman C, Yates BC. Health promotion text blasts for minority adolescent mothers. MCN Am J Matern Child Nurs. 2014 Nov-Dec;39(6):357-62. doi: 10.1097/NMC.0000000000000081. PMID: 25333802.

Bula AK, McCourt C, Magadi M, et al. P6.015 Exploring Experiences with Community Based Promotion of Exclusive Breastfeeding in the Context of HIV in the Rural Malawi

Sexually Transmitted Infections. 2013;89:A374.

Chang SM, Rowe J, Goopy S. Non-family support for breastfeeding maintenance among career women in Taiwan: a qualitative study. Int J Nurs Pract. 2014 Jun;20(3):293-301. doi: 10.1111/ijn.12148. Epub 2013 Aug 15. PMID: 24889002.

Chaput KH, Nettel-Aguirre A, Musto R, Adair CE, Tough SC. Breastfeeding difficulties and supports and risk of postpartum depression in a cohort of womenwho have given birth in Calgary: a prospective cohort study. CMAJ Open. 2016 Mar 21;4(1):E103-9. doi: 10.9778/cmajo.20150009. PMID: 27280109; PMCID: PMC4866929.

Chin NP, Cuculick J, Starr M, Panko T, Widanka H, Dozier A. Deaf mothers and breastfeeding: do unique features of deaf culture and language support breastfeeding success? J Hum Lact. 2013 Nov;29(4):564-71. doi: 10.1177/0890334413476921. Epub 2013 Mar 14. PMID: 23492762; PMCID: PMC4112581.

Condon LJ, Ingram JC. Increasing support for breastfeeding: What can Children's Centres do? Health & Social Care in the Community 2011, 19(6):617 – 625

de Oliveira MI, Souza IE, dos Santos EM, Camacho LA. Avaliação do apoio recebido para amamentar: significados de mulheres usuárias de unidades básicas de saúde do Estado do Rio de Janeiro [Evaluation of breastfeeding support: meanings from mothers receiving care at primary health care units in the State of Rio de Janeiro]. Cien Saude Colet. 2010 Mar;15(2):599-608. Portuguese. doi: 10.1590/S1413-81232010000200036. PMID: 20414627.

Demirtas B, Ergocmen B, Taskin L. Breastfeeding experiences of Turkish women. J Clin Nurs. 2012 Apr;21(7-8):1109-18. doi: 10.1111/j.1365-2702.2011.03848.x. Epub 2011 Sep 20. PMID: 21929556.

Demirtas B. Strategies to support breastfeeding: a review. Int Nurs Rev. 2012 Dec;59(4):474-81. doi: 10.1111/j.1466-7657.2012.01017.x. Epub 2012 Jul 12. PMID: 23134130.

Demirtas B. Multiparous mothers: Breastfeeding support provided by nurses. Int J Nurs Pract. 2015 Oct;21(5):493-504. doi: 10.1111/ijn.12353. Epub 2014 May 9. PMID: 24810711.

Dykes F, Moran VH, Burt S, Edwards J. Adolescent mothers and breastfeeding: experiences and support needs--an exploratory study. J Hum Lact. 2003 Nov;19(4):391-401. doi: 10.1177/0890334403257562. PMID: 14620453.

Fadnes LT, Engebretsen IM, Moland KM, Nankunda J, Tumwine JK, Tylleskär T. Infant feeding counselling in Uganda in a changing environment with focus on the general population and HIV-positive mothers - a mixed method approach. BMC Health Serv Res. 2010 Sep 6;10:260. doi: 10.1186/1472-6963-10-260. PMID: 20815932; PMCID: PMC2944269.

Finigan V. Providing breastfeeding support to ethnically diverse groups of mothers. Prof Nurse. 2003 May;18(9):524-8. PMID: 12764961.

Foley W, Schubert L, Denaro T. Breastfeeding experiences of Aboriginal and Torres Strait Islander mothers in an urban setting in Brisbane. Breastfeed Rev. 2013 Nov;21(3):53-61. PMID: 24592517.

Fraser R. Breast-feeding support in a neonatal surgical unit. Nurs Times. 1997 Nov 19-25;93(47):54-6. PMID: 9418515.

Graffy J, Taylor J. What information, advice, and support do women want with breastfeeding? Birth. 2005 Sep;32(3):179-86. doi: 10.1111/j.0730-7659.2005.00367.x. PMID: 16128971.

Geller S, Yagil Y, Biriotti S, Neufeld MY. Breastfeeding with epilepsy: mothers' experiences and the role of professionals, family, and friends. Breastfeed Med. 2013 Aug;8(4):424-5. doi: 10.1089/bfm.2012.0117. Epub 2013 Jun 7. PMID: 23746207.

Grassley J, Eschiti V. Grandmother breastfeeding support: what do mothers need and want? Birth. 2008 Dec;35(4):329-35. doi: 10.1111/j.1523-536X.2008.00260.x. PMID: 19036046.

Hall Moran V, Edwards J, Dykes F, Downe S. A systematic review of the nature of support for breast-feeding adolescent mothers. Midwifery. 2007 Jun;23(2):157-71. doi: 10.1016/j.midw.2006.06.005. Epub 2006 Oct 18. PMID: 17052824.

Hauck YL, Fenwick J, Dhaliwal SS, Butt J, Schmied V. The association between women's perceptions of professional support and problems experienced on breastfeeding cessation: a Western Australian study. J Hum Lact. 2011 Feb;27(1):49-57. doi: 10.1177/0890334410386956. Epub 2010 Dec 22. PMID: 21177989.

Hauck YL, Blixt I, Hildingsson I, Gallagher L, Rubertsson C, Thomson B, Lewis L. Australian, Irish and Swedish women's perceptions of what assisted them to breastfeed for six months: exploratory design using critical incident technique. BMC Public Health. 2016 Oct 10;16(1):1067. doi: 10.1186/s12889-016-3740-3. PMID: 27724932; PMCID: PMC5057437.

Heidari Z, Kohan S, Keshvari M. Empowerment in breastfeeding as viewed by women: A qualitative study. J Educ Health Promot. 2017 May 5;6:33. doi: 10.4103/jehp.jehp_34_16. PMID: 28584833; PMCID: PMC5441199.

Hunter-Adams J. Mourning the support of women postpartum: The experiences of migrants in Cape Town, South Africa. Health Care Women Int. 2016 Sep;37(9):1010-1024. doi: 10.1080/07399332.2016.1185106. Epub 2016 May 4. PMID: 27144493.

Isabella PH, Isabella RA. Correlates of successful breastfeeding: a study of social and personal factors. J Hum Lact. 1994 Dec;10(4):257-64. doi: 10.1177/089033449401000421. PMID: 7619281.

Kantrowitz-Gordon I; Abbott S; Hoehn R; Mindfulness Childbirth Classes Transform the Experiences of Postpartum Women...American College of Nurse-Midwives’ 62nd Annual Meeting in May 2017. 10.1111/jmwh.12692

Koerber A, Brice L, Tombs E. Breastfeeding and problematic integration: results of a focus-group study. Health Commun. 2012;27(2):124-44. doi: 10.1080/10410236.2011.571754. Epub 2011 Aug 11. PMID: 21834716.

Laterra A, Ayoya MA, Beaulière JM, Bienfait M, Pachón H. Infant and young child feeding in four departments in Haiti: mixed-method study on prevalence of recommended practices and related attitudes, beliefs, and other determinants. Rev Panam Salud Publica. 2014 Nov;36(5):306-13. PMID: 25604100.

Lin LC, Lee TY, Kuo SC, Mu PF, Shu HQ. [The lived experience of postpartum women receiving rooming-in care]. Hu Li Za Zhi. 2004 Feb;51(1):35-44. Chinese. PMID: 15045891.

McVicar S, Kirkpatrick P. The effectiveness and maternal satisfaction of interventions supporting the establishment of breast-feeding for women from disadvantaged groups: a comprehensive systematic review protocol. JBI Database of Systematic Reviews and Implementation Reports: June 2014 - Volume 12 - Issue 6 - p 420-476. doi: 10.11124/jbisrir-2014-1561

McInnes RJ, Chambers JA. Supporting breastfeeding mothers: qualitative synthesis. J Adv Nurs. 2008 May;62(4):407-27. doi: 10.1111/j.1365-2648.2008.04618.x. PMID: 18476941.

Nelson AM. Maternal-newborn nurses' experiences of inconsistent professional breastfeeding support. J Adv Nurs. 2007 Oct;60(1):29-38. doi: 10.1111/j.1365-2648.2007.04373.x. PMID: 17824937.

Peritore NR, Communicating social support: understanding complexities of breastfeeding communication among African American mothers (2016). Theses and Dissertations--Communication. 53. https://uknowledge.uky.edu/comm_etds/53

Price L. Can early breastfeeding support increase the 6-8 week breastfeeding prevalence rate? Community Pract. 2014 May;87(5):30-3. PMID: 24881195.

Rwami J ; Muheki M ; Francis K ; Wood C ; The value of providing an antenatal and postnatal support group for HIV-positive women. 2009. http://dx.doi.org/10.1111/j.1468-1293.2009.00706.x

Segeel IB, du Plessis D.The childbirth and breastfeeding experiences of primigravidas who attended childbirth education classes. 2016.Health SA Gesondheid | Vol 11, No 2 | a223 | DOI: https://doi.org/10.4102/hsag.v11i2.223 |

Serçekuş P, Mete S. Turkish women's perceptions of antenatal education. Int Nurs Rev. 2010 Sep;57(3):395-401. doi: 10.1111/j.1466-7657.2009.00799.x. PMID: 20796072.

Sim TF, Hattingh HL, Sherriff J, Tee LB. What Do Breastfeeding Women Taking Herbal Galactagogues Perceive of Community Pharmacists' Role in Breastfeeding Support? A Qualitative Study. Int J Environ Res Public Health. 2015 Sep 8;12(9):11132-45. doi: 10.3390/ijerph120911132. PMID: 26371023; PMCID: PMC4586665.
