# Supplementary material for: Factors that influence women's engagement with breastfeeding support: A qualitative evidence synthesis
Source: Matern Child Nutr. 2022 Aug 25;18(4):e13405. doi: 10.1111/mcn.13405 (PMC9480951; doi:10.1111/mcn.13405)
Supplement: Supplementary file 4 — Supplementary information. [file MCN-18-e13405-s005.docx]

### Data extraction user guide

| **Study characteristic** | **Description** | **Relevant example** | |
| --- | --- | --- | --- |
| **Columns A** Study |  |  | |
| **Columns B** Language |  |  | |
| **Columns C** Country of study | Please indicate the geographical location the study was conducted in:  ...........................................................................................................  Please indicate whether the study takes place in:   - Urban region - Rural region - Other: ……………………………………………………………………………………….. - Unsure - No information |  | |
| **Columns D** Low- or middle- or high-income country | Please indicate:   - Low-income - Middle-income - High-income   OESO recognizes the following countries as developing country (please follow the [link](http://www.cmo.nl/nothingaboutus/index.php/ontwikkelingssamenwerking/landenlijst) for the classification).   - Other: ……………………………………………………………………………………….. - Unsure - No information |  | |
| **Columns E** External contextual factors | Can you provide a description of external contextual factors? This could include political, economic, religious, cultural or political context:  ...........................................................................................................   - Unsure - No information |  | |
| **Columns F** Theory | What is the theory underlying the intervention?  Theoretical or conceptual framework, programme theory or change model, theoretical lens, etc... Please describe:  ...........................................................................................................   - Other: ……………………………………………………………………………………….. - Unsure - No information | the basic paradigms used in research (like interpretivism, critical theory, constructivism, constructivism, poststructuralism, positivism, realism, empowerment theory etc.) | |
| **Columns G** Objective of the study | What is the purpose of the qualitative investigation?  ...........................................................................................................   - Unsure - No information |  | |
| **Columns H** Significance of the study | Do the authors describe the importance of the study in terms of   - Scientific value - Value for society - Value for professional practice - Value for policy - Other: ……………………………………………………………………………………….. - Unsure - No information |  | |
| **Columns I**  Study design | What research design was applied?   - Grounded theory - Phenomenological studies - Narrative studies - Action research studies - Case studies - Visual studies - Other: ……………………………………………………………………………………….. - Unsure - No information |  | |
| **Columns J** Data collection techniques | What research methods were used to collect the data?   - Focus groups - Face to face interviews - Observations - Arts and design based methods - Literature study - Secondary data from other studies - Evaluation study - Other: ……………………………………………………………………………………….. - Unsure - No information |  | |
| **Columns K** Data analysis techniques | What methods were used to analyse the data?   - Content analysis - Thematic analysis - Constant comparison - Visual analysis - Other qualitatively inspired analytical approaches: ………………… - Unsure - No information: |  | |
| **Columns L** Participant group | Describe the participant group (including personal/external characteristics):   - Women who give birth to one child - Women who give birth to multiple children - First-time mother - Women with specific health conditions (HIV, cancer, etc.) - Women of babies with special needs (multiple birth, babies with disabilities, premature infants) - Women in employment (part- or fulltime)   In reference to Cochrane [EQUITY PROGRESS PLUS](http://methods.cochrane.org/equity/projects/evidence-equity/progress-plus), we are also interested in the following characteristic. Please describe them where possible:  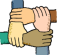  Race/ethnicity/culture/language  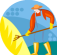  Occupation  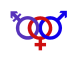Gender/sex  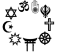  Religion  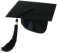  Education  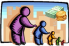 Socioeconomic status  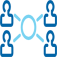  Social capital   - Other: ……………………………………………………………………………………….. - Unsure - No information |  | |
| **Columns M** Implementers of support | Who are the implementers?   - One group or type - More than one group or type - Other: ……………………………………………………………………………………….. - Unsure - No information | *Peer group support or individuals (e.g. lactation consultant, midwife, etc.)* |  |
| **Columns N** Characteristic of the implementing organisation, network or association | Are any characteristics of the implementers considered?  Again, please bear the Cochrane [EQUITY PROGRESS PLUS](http://methods.cochrane.org/equity/projects/evidence-equity/progress-plus) in mind.   - Occupation:   - Lay   - Degree - Years of experience: ………………………………………………………………… - Competence: …………………………………………………………………………… - Ethnicity: ………………………………………………………………………………… - Age: ………………………………………………………………………………………… - Gender: ……………………………………………………………………………………. - Religion: …………………………………………………………………………………… - Other: ……………………………………………………………………………………….. - Unsure - No information |  |  |
| **Columns O** Associate organisations and community partners | Does the programme involve partnerships or collaborations? Where relevant, extract data on who the partners are:  ...........................................................................................................   - Other: ……………………………………………………………………………………….. - Unsure - No information | *The implementers work together with a local community leader to increase acceptance of the intervention.* |  |
| **Columns P** Type of support offered | These could include:   - Face- to-face-(or one-on-on) support - Group sessions - Educational sessions (e.g. in the form of a group meeting) - Information provision (information given via the phone) - Intervention in case of (urgent) medical issues - Home visiting programmes - Peer (education) programmes - Clinic appointments - Other: ……………………………………………………………………………………….. - Unsure - No information | *Face-to-face support by a midwife in the case of latching difficulties. Phone call with a lactation consultant in the case of mastitis or lactogenesis.*  *Clinic appointments that are specifically aimed at imparting knowledge about breastfeeding and could involve prospective fathers or not.* |  |
| **Columns Q** Multiple or single component interventions | Does the intervention include more than one intervention strategy, element, activity or component?   - Single intervention: …………………………………………………………………… - Multiple intervention: ………………………………………………………………. - Other: ……………………………………………………………………………………….. - Unsure - No information | *A breastfeeding support programme based on home visits combined with weekly phone text messages containing breastfeeding information.*  *Interventions could also include breastfeeding education session, printed information, video, peer counselling and lactation consultation.* |  |
| **Columns R** Form of communication of support | The form of communication has to happen within an interaction in term of human contact! These could include:   - Verbal - Written - Oral - Visual - Electronic communication - Other: ……………………………………………………………………………………….. - Unsure - No information | *A midwife handing over a brochure and reading it through together with the mother. Reading materials, either online or in print.* |  |
| **Columns S** Breastfeeding phase | Tick in which phase the support was given.   - Initiation i.e. from birth up to? x days/weeks): …………………………. - Duration (from X to y): ……………………………………………………………… - Both: …………………………………………………………………………………………. - Other: ……………………………………………………………………………………….. - Unsure - No information |  |  |
| **Columns T** Setting of the support intervention | - Setting in which the support was offered/ implemented in. - One setting: ………………………………………………………………………………. - More settings: …………………………………………………………………………… - Other: ……………………………………………………………………………………….. - Unsure - No information | *Settings could include health facilities, home based interventions, facilities of local support communities or home visits.* |  |
| **Columns U** Dose of support | - Frequency: ………………………………………………………………………………… - and/or duration: ……………………………………………………………………….. - and/ or intensity: ………………………………………………………………………. - of the support intervention. Specify if multiple aspects assessed! - Other: ……………………………………………………………………………………….. - Unsure - No information |  |  |
| **Columns V** Concepts deriving | Please describe:  ...........................................................................................................   - Unsure - No information |  |  |
| **Columns W** Main conclusion | What is the main conclusion of the study? Please specify in max. 5 phrases.  ...........................................................................................................  ...........................................................................................................  ...........................................................................................................  ...........................................................................................................  ........................................................................................................... |  |  |
