# Supplementary material for: Factors that influence women's engagement with breastfeeding support: A qualitative evidence synthesis
Source: Matern Child Nutr. 2022 Aug 25;18(4):e13405. doi: 10.1111/mcn.13405 (PMC9480951; doi:10.1111/mcn.13405)
Supplement: Supplementary file 5 — Supplementary information. [file MCN-18-e13405-s004.docx]

## Summary of Qualitative Findings tables

Table 5 Summary of Qualitative Findings table I

| **Summary of review finding** | **Studies contributing to the review finding** | **GRADE‐CERQual assessment of confidence in the evidence** | **Explanation of GRADE‐CERQual assessment** |
| --- | --- | --- | --- |
| **Process factors affecting implementation: INFORMATION PROVISION - THE WHAT** | | | |
| ***Elements that should be included in breastfeeding information and messages*** | | | |
| **Finding 1:** Women do not want technical breastfeeding information | Craig 2010; Cripe 2010; Rossman 2010; Thomson 2012 | **Moderate confidence** | Due to minor concerns regarding methodological limitations and relevance and moderate concerns regarding coherence and adequacy. |
| **Finding 2:** Women want consistent messages about infant feeding | Ahluwalia 2000, Beake 2005; Bula 2015, Condon 2012, Cripe 2010, Cross-Barnet 2012; Engstrom 2000; Fox 2015, Hong 2003; Islam 2016; Meier 2007; Noble-Carr 2012 | **High confidence** |  |
| **Finding 3:** Women want realistic information of benefits as well as risks and challenges that might be faced in relation to breastfeeding | Battersby 2002, Breedlove 2005, Bula 2015, Condon 2012, Da Rocha 2013, Fox 2015; Meier 2007; Noble-Carr 2012; Thomson 2012 | **Moderate confidence** | Due to moderate concerns about methodological limitations and coherence and minor concerns regarding relevance and adequacy. |

Table 6 Evidence Profile I

| **Finding 1: Women do not want technical breastfeeding information** |
| --- |
| **Assessment for each GRADE-CERQual component** |
| *Methodological limitations: Minor to moderate concerns regarding methodological limitations due to poor reporting from one study on most of the categories.* |
| *Coherence: Moderate concerns for coherence du to data not explicitly supporting the review finding.* |
| *Relevance: Minor concerns regarding relevance due to partial relevance due to a narrow range of settings (high-income countries only).* |
| *Adequacy: Moderate concerns regarding adequacy due to a small number of contributing studies in the context of the specific review finding.* |
| **Overall GRADE-CERQual assessment and explanation**  **Moderate confidence:** Due to minor concerns regarding methodological limitations and relevance and moderate concerns regarding coherence and adequacy. |
| **Contributing studies** |
| Craig 2010; Cripe 2010; Rossman 2010; Thomson 2012 |
| **Finding 2: Women want consistent messages about infant feeding** |
| **Assessment for each GRADE-CERQual component** |
| *Methodological limitations: Moderate to major concerns regarding methodological limitations due to poor reporting from seven studies on context, sampling, methods researcher reflexivity and ethics.* |
| *Coherence: No concerns regarding coherence.* |
| *Relevance: Minor concerns regarding relevance due to partial relevance due to a narrow range of settings.* |
| *Adequacy: No to minor concerns regarding adequacy.* |
| **Overall GRADE-CERQual assessment and explanation**  **High confidence** |
| **Contributing studies** |
| Ahluwalia 2000, Beake 2005; Bula 2015, Condon 2012, Cripe 2010, Cross-Barnet 2012; Engstrom 2000; Fox 2015, Hong 2003; Islam 2016; Meier 2007; Noble-Carr 2012 |
| **Finding 3: Women want realistic information of benefits as well as risks and challenges of breastfeeding** |
| **Assessment for each GRADE-CERQual component** |
| *Methodological limitations: Moderate to major concerns regarding methodological limitations due to poor reporting on almost all criteria in more than half of the studies.* |
| *Coherence: Moderate concerns regarding coherence due to partial data not explicitly supporting the review finding.* |
| *Relevance: Minor concerns regarding relevance due to partial relevance due to a narrow range of settings (predominantly high-income countries).* |
| *Adequacy: Minor concerns regarding adequacy due to sufficient numbers of contributing studies.* |
| **Overall GRADE-CERQual assessment and explanation**  **Moderate confidence**: Moderate concerns about methodological limitations and coherence and minor concerns regarding relevance and adequacy. |
| **Contributing studies** |
| Battersby 2002, Breedlove 2005, Condon 2012, Fox 2015; Meier 2007; Noble-Carr 2012; Thomson 2012 |

Table 7 Summary of Qualitative Findings table II

| **Summary of review finding** | **Studies contributing to the review finding** | | **GRADE‐CERQual assessment of confidence in the evidence** | **Explanation of GRADE‐CERQual assessment** | |
| --- | --- | --- | --- | --- | --- |
| **Process factors affecting implementation: TYPE OF IMPLEMENTERS – THE WHOM** | | | | | |
| ***(Un-)Supportive characteristics of implementers of breastfeeding support*** | | | | | |
| **Finding 4:** Women prefer the support of an implementer who has gone through similar experiences in relation to breastfeeding | Cripe 2010, Rossman 2010, Thomson 2012 | | **Moderate confidence** | Due to minor concerns about methodological limitations and relevance and moderate concerns regarding adequacy. | |
| **Finding 5:** Women experienced disconnected encounters with hospital staff | Ahluwalia 2012, Beake 2005, Bula 2015, Condon 2012, Fox 2015, Hong 2003 | | **High confidence** |  | |
| **Finding 6:** Women value one-on-one support in the form of (online) community-based supporters | Battersby 2002, Breedlove 2005, Bridges 2016, Cripe 2010, Fox 2015, Leahy-Warren 2017 | | **Moderate confidence** | Due to moderate to major concerns regarding methodological limitations and minor concerns about coherence, relevance and adequacy. | |
| **Finding 7:** Women judge the quality of the information provided as high when delivered in the context of official breastfeeding support programmes | | Bridges 2016, Bula 2015, Noble-Carr 2012, Thomson 2012 | **High confidence** |  |  |

Table 8 Evidence Profile II

| **Finding 4: Women prefer the support of an implementer who has gone through similar experiences in relation to breastfeeding** |
| --- |
| **Assessment for each GRADE-CERQual component** |
| *Methodological limitations: Minor concerns regarding methodological limitations.* |
| *Coherence: No concerns regarding coherence.* |
| *Relevance: Minor concerns regarding relevance due to partial relevance as studies are from a narrow range of settings (high-income countries only).* |
| *Adequacy: Moderate concerns regarding adequacy due to a small number of contributing studies.* |
| **Overall GRADE-CERQual assessment and explanation**  **Moderate confidence:** Due to minor concerns about methodological limitations and relevance and moderate concerns regarding adequacy. |
| **Contributing studies** |
| Cripe 2010; Rossman 2010; Thomson 2012 |
| **Finding 5: Women experienced disconnected encounters with hospital staff** |
| **Assessment for each GRADE-CERQual component** |
| *Methodological limitations: Moderate to major concerns regarding methodological limitations of four contributing studies on all criteria.* |
| *Coherence: No concerns regarding coherence.* |
| *Relevance: Minor concerns regarding relevance (a variety of settings and implementers and setting of intervention; but only one low-income country).* |
| *Adequacy: No concerns regarding adequacy.* |
| **Overall GRADE-CERQual assessment and explanation**  **High confidence:** Due to moderate concerns regarding methodological limitations and minor concerns about relevance |
| **Contributing studies** |
| Ahluwalia 2012, Beake 2005, Bula 2015, Condon 2012, Fox 2015, Hong 2003 |
| **Finding 6: Women value one-on-one support in the form of (online) community-based supporters** |
| **Assessment for each GRADE-CERQual component** |
| *Methodological limitations: Moderate to major concerns regarding methodological limitations of three contributing studies on all criteria.* |
| *Coherence: Minor concerns regarding coherence due to two studies reporting little data on this finding.* |
| *Relevance: Minor concerns regarding relevance (a variety of settings and implementers and setting of intervention; but no low-income country).* |
| *Adequacy: Minor concerns regarding adequacy due to a partial thinness of data.* |
| **Overall GRADE-CERQual assessment and explanation**  **Moderate confidence:** Due to moderate to major concerns regarding methodological limitations and minor concerns about coherence, relevance and adequacy. |
| **Contributing studies** |
| Battersby 2002, Breedlove 2005, Bridges 2016, Cripe 2010, Fox 2015, Leahy-Warren 2017 |
| **Finding 7: Women judge the quality of the information provided as high when delivered in the context of official breastfeeding support programmes** |
| **Assessment for each GRADE-CERQual component** |
| *Methodological limitations: Minor concerns regarding methodological limitations.* |
| *Coherence: No to minor concerns regarding coherence.* |
| *Relevance: Minor concerns regarding relevance due to partial relevance due to a narrow range of settings.* |
| *Adequacy: No to minor concerns regarding adequacy due to rich data across studies, despite the number of studies is small.* |
| **Overall GRADE-CERQual assessment and explanation**  **High confidence** |
| **Contributing studies** |
| Bridges 2016, Bula 2015, Noble-Carr 2012, Thomson 2012 |
|  |

Table 9 Summary of Qualitative Findings table III

| **Summary of review finding** | **Studies contributing to the review finding** | **GRADE‐CERQual assessment of confidence in the evidence** | **Explanation of GRADE‐CERQual assessment** |
| --- | --- | --- | --- |
| **Process factors affecting implementation: (NON) OPTIMAL DELIVERY MODES – THE HOW** | | | |
| ***Being supported as an individual*** | | | |
| **Finding 8:** Women want implementers of support to respect their individual choice whether and how to breastfeed | Ahluwalia 2000, Andreson 2013, Battersby 2002, Breedlove 2005, Bula 2015, Condon 2012, Craig 2010. Cripe 2010, Engstrom 2000, Fox 2015, Meier 2007, Noble-Carr 2012, Rossman 2010, Thomson 2012 | **Moderate confidence** | Due to moderate to major concerns of methodological limitations, moderate concerns about coherence and minor concerns about relevance and adequacy. |
| **Finding 9:** Women do not like to be touched at the breast | Noble-Carr 2012, Weimers 2006 | **Low confidence** | Due to minor concerns regarding methodological limitations and coherence, moderate concerns about relevance and major concerns about adequacy. |

Table 10 Evidence Profile III

| **Finding 8: Women want implementers of support to respect their individual choice whether and how to breastfeed** |
| --- |
| **Assessment for each GRADE-CERQual component** |
| *Methodological limitations: Moderate to major concerns regarding methodological limitations due to poor reporting from six studies on all criteria.* |
| *Coherence: Moderate concerns regarding coherence as not all data from the included studies contribute to the findings sufficiently.* |
| *Relevance: No to minor concerns regarding relevance.* |
| *Adequacy: Minor concerns regarding adequacy due to a large number of contributing studies.* |
| **Overall GRADE-CERQual assessment and explanation**  **Moderate confidence:** Due to moderate to major concerns of methodological limitations, moderate concerns about coherence and minor concerns about relevance and adequacy. |
| **Contributing studies** |
| Ahluwalia 2000, Andreson 2013, Battersby 2002, Breedlove 2005, Bula 2015, Condon 2012,Craig 2010, Cripe 2010, Engstrom 2000, Fox 2015, Islam 2016, Meier 2007, Noble-Carr 2012, Rossman 2010, Thomson 2012 |
| **Finding 9: Women do not like their breasts be touched** |
| **Assessment for each GRADE-CERQual component** |
| *Methodological limitations: Minor concerns regarding methodological limitations.* |
| *Coherence: No to minor concerns regarding coherence.* |
| *Relevance: Moderate concerns regarding relevance due to partial relevance due to a narrow range of settings (high-income countries only).* |
| *Adequacy: Major concerns regarding adequacy due to only partial data explicitly supporting the review finding and small number of contributing studies.* |
| **Overall GRADE-CERQual assessment and explanation**  **Low confidence:** Due to minor concerns regarding methodological limitations and coherence, moderate concerns about relevance and major concerns about adequacy. |
| **Contributing studies** |
| Noble-Carr 2012, Weimers 2006 |
|  |

Table 11 Summary of Qualitative Findings table IV

| **Summary of review finding** | **Studies contributing to the review finding** | **GRADE‐CERQual assessment of confidence in the evidence** | **Explanation of GRADE‐CERQual assessment** |
| --- | --- | --- | --- |
| **CARE PATHWAYS – THE WHERE AND WHEN** | | | |
| ***Service designs*** | | | |
| **Finding 10:** Women want support to be easily and flexibly available | Battersby 2002, Beake 2005, Bridges 2016, Bula 2015, Fox 2015, Hong 2003, Noble-Carr 2012, Thomson 2012 | **Moderate confidence** | Due to minor to moderate concerns regarding methodological limitations and coherence and minor concerns regarding relevance and adequacy. |
| **Finding 11:** Women perceive benefits of home visits in combining various forms of support | Bula 2015, Rossman 2010, Thomson 2012 | **Moderate confidence** | Due to moderate concerns regarding coherence and adequacy and no to minor concerns about methodological limitations and relevance. |

Table 12 Evidence Profile IV

| **Finding 10: Women want support to be easily and flexibly available** |
| --- |
| **Assessment for each GRADE-CERQual component** |
| *Methodological limitations: Minor to moderate concerns regarding methodological limitations due to poor reporting from three studies.* |
| *Coherence: Minor to moderate concerns regarding coherence due to not all data directly supporting the review finding.* |
| *Relevance: Minor concerns regarding relevance (a variety of settings and implementers and setting of intervention and geographic regions).* |
| *Adequacy: Minor concerns regarding adequacy due to partial data not explicitly contributing to the review finding and high number of contributing studies.* |
| **Overall GRADE-CERQual assessment and explanation**  **Moderate confidence:** Due to minor to moderate concerns regarding methodological limitations and coherence and minor concerns regarding relevance and adequacy. |
| **Contributing studies** |
| Battersby 2002, Beake 2005, Bridges 2016, Bula 2015, Fox 2015, Hong 2003, Thomson 2012, Noble-Carr 2012 |
| **Finding 11: Women perceive benefits of home visits in combining various forms of support** |
| **Assessment for each GRADE-CERQual component** |
| *Methodological limitations: No to minor concerns regarding methodological limitations.* |
| *Coherence: Moderate concerns regarding coherence due to some disconfirming data presented by one study.* |
| *Relevance: Minor to moderate concerns regarding relevance due to a limited group of implementers of support.* |
| *Adequacy: Moderate concerns regarding adequacy due to a small number of contributing studies supporting the review finding.* |
| **Overall GRADE-CERQual assessment and explanation**  **Low confidence**: Due to moderate concerns regarding coherence and adequacy and minor concerns about methodological limitations and relevance. |
| **Contributing studies** |
| Bula 2015, Rossman 2010, Thomson 2012 |

Table 13 Summary of Qualitative Findings table V

| **Summary of review finding** | **Studies contributing to the review finding** | **GRADE‐CERQual assessment of confidence in the evidence** | **Explanation of GRADE‐CERQual assessment** | |
| --- | --- | --- | --- | --- |
| **CARE PATHWAYS** | | | | |
| ***Care pathway timeline*** | | | | |
| **Finding 12:** Women want information about breastfeeding support options early on in their pregnancy | Craig 2010, Fox 2015, Islam 2016, Hong 2003, Noble-Carr 2012 | **Low confidence** | Due to major concerns about methodological limitations, minor concerns regarding relevance and moderate concerns regarding coherence and adequacy. | |
| **Finding 13:** Women want continuity | Battersby 2002, Breedlove 2005, Bula 2015, Craig 2010, Fox 2015, Rossman 2010, Thomson 2012 | **Moderate confidence** | Due to moderate concerns regarding methodological limitations and minor concerns about coherence, relevance and adequacy. |  |
| **Finding 14:** Women perceive the optimal duration of physical support as the observation of whole feeds | Bula 2015, Condon 2012, Hong 2003 | **Low confidence** | Due to moderate concerns about methodological limitations, coherence and adequacy and minor concerns regarding relevance. | |

Table 14 Evidence Profile V

| **Finding 12: Women want information about breastfeeding support options in early pregnancy** | |
| --- | --- |
| **Assessment for each GRADE-CERQual component** | |
| *Methodological limitations:* Major concerns regarding methodological limitations due to poor reporting from all studies on all criteria. | |
| *Coherence: Moderate concerns regarding coherence due to data not explicitly supporting the review finding.* | |
| *Relevance: Minor concerns regarding relevance due to partial relevance due to a narrow range of settings (high-income countries only).* | |
| *Adequacy: Moderate concerns regarding adequacy due to a small number of contributing studies in the context of this finding.* | |
| **Overall GRADE-CERQual assessment and explanation** | |
| **Low confidence**: Due to major concerns about methodological limitations, minor concerns regarding relevance and moderate concerns regarding coherence and adequacy. | |
| **Contributing studies** | |
| Craig 2010, Fox 2015, Islam 2016, Hong 2003, Noble-Carr 2012 | |
| **Finding 13: Women want continuity in breastfeeding support** |  |
| **Assessment for each GRADE-CERQual component** |  |
| *Methodological limitations: Moderate to major concerns regarding methodological limitations due to poor reporting from three studies on all categories.* |  |
| *Coherence: No to minor concerns regarding coherence.* |  |
| *Relevance: Minor concerns regarding relevance due to a narrow range of settings.* |  |
| *Adequacy: Minor concerns regarding adequacy due to two studies contributing with thin data.* |  |
| **Overall GRADE-CERQual assessment and explanation**  **Moderate confidence:** Due to moderate concerns regarding methodological limitations and minor concerns about coherence, relevance and adequacy |  |
| **Contributing studies** |  |
| Battersby 2002, Breedlove 2005, Bula 2015, Craig 2010, Fox 2015, Rossman 2010, Thomson 2012 |  |
| **Finding 14: Women perceive the optimal duration of physical support as the observation of whole feeds** |  |
| **Assessment for each GRADE-CERQual component** |  |
| *Methodological limitations: Moderate concerns regarding methodological limitations due to poor reporting from one study on most of the categories.* |  |
| *Coherence: Moderate concerns regarding coherence due to one disconfirming case.* |  |
| *Relevance: Minor concerns regarding relevance due to partial relevance of settings and implementers of support.* |  |
| *Adequacy: Moderate concerns regarding adequacy due a small number of contributing studies within the context of the review finding.* |  |
| **Overall GRADE-CERQual assessment and explanation**  **Low confidence**: Due to moderate concerns about methodological limitations, coherence and adequacy and minor concerns regarding relevance. |  |
| **Contributing studies** |  |
| Bula 2015, Condon 2012, Hong 2003 |  |
